# Structural chemistry of intermetallic compounds for active site design in heterogeneous catalysis

**DOI:** 10.1039/d5sc01810b

**Published:** 2025-04-21

**Authors:** Nilanjan Roy, Kathryn MacIntosh, Mustafa Eid, Griffin Canning, Robert M. Rioux

**Affiliations:** a Department of Chemical Engineering, Pennsylvania State University University Park PA 16802 USA rmr189@psu.edu; b Department of Chemistry, Pennsylvania State University University Park PA 16802 USA

## Abstract

Completely or partially ordered intermetallic compounds possess unique electronic structure and chemical bonding, establishing them as an emergent class of catalytic materials for selective hydrogenation reactions. In this review, we focus on the structural and chemical aspects of different classes of intermetallic compounds, followed by illustrative examples highlighting the impact of their structural/chemical features on catalytic hydrogenation. We limit the scope of our discussion to the selective hydrogenation of alkynes (acetylene). We focus our discussion on how the isolation of active sites, formation of defined surface ensembles, partial charge transfer between heteroatoms, and alteration of the surface electronic structure impact activity and selectivity toward the desired product(s), based on recent literature observations. This review contributes to informing the appropriate selection of intermetallic catalysts for hydrogenation reactions to achieve high selectivity.

## Abstract 2

Completely or partially ordered intermetallic compounds possess unique geometric ensembles, electronic structure and chemical bonding scenarios, establishing them as an emergent class of catalytic materials for selective hydrogenation reactions.

## Graphical abstract

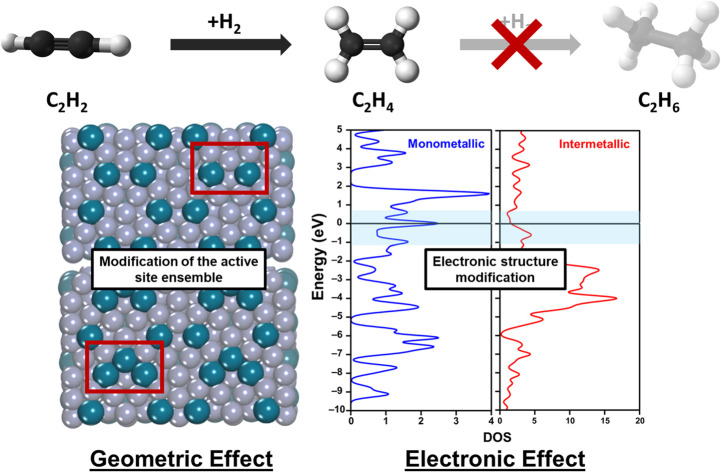

## 1. Introduction

The well-defined and ordered structures of intermetallic compounds offer the opportunity to rationally design catalysts with specific active sites for various catalytic processes. The precise control over active ensembles establishes intermetallic compounds well-suited as model catalysts for gaining a deeper understanding of structure–activity–selectivity relationships. Comprehensive reviews on the broad field of intermetallic compounds in heterogeneous catalysis are already available in the literature.^1–11^ However, the correlation between the structural chemistry of intermetallic compounds and their application as model catalysts in heterogeneous catalysis is still lacking.

The selective hydrogenation of acetylene is a catalytic process of significant industrial importance, as the production of polyethylene requires that acetylene impurities in the ethylene stream be reduced to less than 5–10 ppm.^12,13^ Polymerization catalysts are extremely sensitive to the presence of alkynes, with even low levels causing significant poisoning and loss of activity. Therefore, highly selective catalysts are required to hydrogenate low concentrations of alkynes in the presence of a large excess of alkenes while minimizing over-hydrogenation to the undesired alkane.

In this review, we introduce the ‘structural chemistry’ of intermetallic compounds and discuss how their structural features can be leveraged to develop selective and active catalysts for hydrogenation reactions. This article is broadly divided into two parts: part I discusses the fundamental aspects of intermetallic compounds, such as their definition, structural properties (including bulk and surface characterization), and their unique electronic structure and chemical bonding scenarios. Part II focuses on recent investigations into the selective hydrogenation of acetylene using intermetallics as model catalysts, with particular emphasis on the precise control over active site configuration and composition inherent to intermetallic compounds.

## 2. Part I: fundamental aspects of intermetallic compounds

### 2.1 Definitions: solid solutions *vs.* intermetallic compounds

The mixing of metals has played an important role in human society. For thousands of years, it has been known that combining hard and soft metals at elevated temperatures results in the formation of new materials with improved properties. Early examples include bronze (88% Cu + 12% Sn) and brass (Cu + Zn), with early steel variants emerging a couple thousand years later.^14,15^ These materials exhibit emergent properties relative to the constituent metals. These new materials and their corresponding emergent properties revolutionized human society at the time, and the discovery of new metal mixtures continues to drive technological advancement today. For example, Ni–Al based intermetallic compounds are widely used in furnace components, jet engines, power plants, and water turbines due to their significantly high strength, low density, excellent thermal conductivity, and resistance to corrosion and oxidation.^16–18^ Increasingly complex mixtures of a wide array of elements have been discovered with properties desirable for different applications, including heterogeneous catalysis. The optimization of these materials for catalytic applications requires an understanding of the fundamental principles behind their formation and structure–property behavior.

#### 2.1.1 Metal alloys/solid solutions

**Fig. 1:**
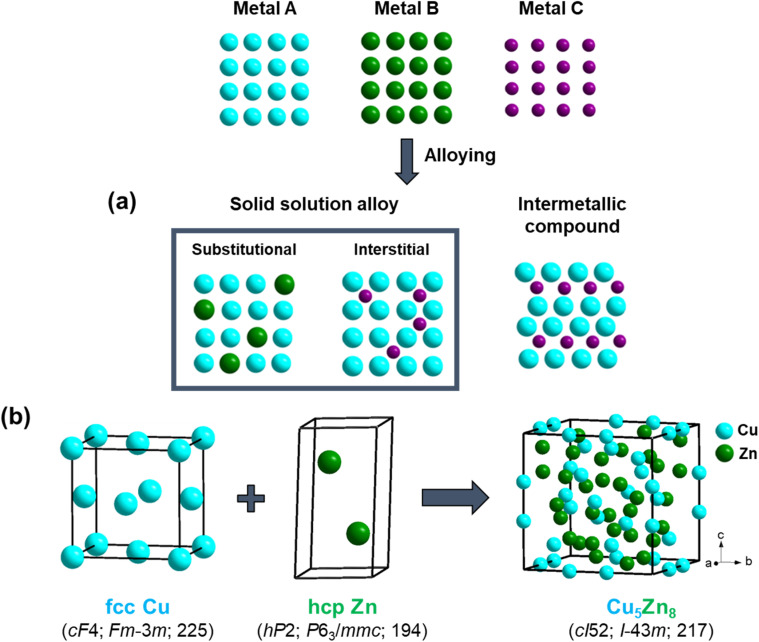
(a) Representative average structures of solid solutions and intermetallic compounds. In solid solutions, the structure type remains the same as at least one of the constituents, which distinguishes them from intermetallic compounds. (b) Structure of ideal γ-brass Cu_5_Zn_8_ (bcc),^19^ which differs from elemental Cu (fcc) and Zn (hcp), representing an intermetallic compound. All structural figures in this manuscript were drawn using DIAMOND 3 (ref. 20) and CIF files were collected from Pearson's Crystal Database.^21^

Metal mixtures can be broadly classified into two categories: (i) alloys and (ii) intermetallic compounds. In the case of alloys, one metal (minor component) is randomly distributed in the matrix of the other metal (major component) and the structure type of the overall system is the same as the major component (Fig. 1). For example, when 2% Zn (hcp) diffuses into 98% (fcc) Cu, the overall structure remains the same as that of Cu; Zn substitutes some Cu positions randomly. In other cases, the minor component occupies interstitial positions randomly (*e.g.*, C occupies the interstitial positions of bcc-Fe in steel)^22^ in the structure of the major component. These are referred to as substitutional solid solutions and interstitial solid solutions, respectively. The formation of solid solutions is primarily guided by a set of rules provided by W. Hume Rothery.^23–25^

(a) The atomic size of the solute (minor component) in substitutional solid solutions should differ by less than 15% from that of the solvent (major component).

(b) For an interstitial solid solution, the solute atom's radius shouldn't be greater than 59% of the solvent.

(c) Electronegativities and valence electron counts (VEC) of the components must be comparable for the formation of solid solutions, whether substitutional or interstitial.

#### 2.1.2 Intermetallic compounds

Intermetallic compounds (IMCs) comprise two or more metals (or metalloids) at a stoichiometric ratio within a specific range and, contrary to solid solutions, adopt a different crystal structure than the constituents (Fig. 1). When referred to as ‘intermetallic alloys’, they should not be confused with random alloys. A key difference between alloys and intermetallic compounds is the way the constituent atoms form bonds. In alloys, the bonding electrons are delocalized throughout the material, indicating that bonding is predominantly non-directional. Bonds in intermetallic compounds possess directional, covalent, or even in some cases, ionic character.^26^ Directional bonding in intermetallic compounds leads to a strongly modified electronic structure compared with the pure constituent elements, which results in significant differences in various chemical and physical properties. These changes open possibilities for the application of IMCs in heterogeneous catalysis,^27,28^ atomic hydrogen storage,^29^ thermoelectrics,^30^ superconductors,^31,32^ and magnetic materials.^33^ Ultimately, the unique combination of covalent and ionic interactions alongside the presence of conducting free electrons leads to appealing combinations of crystallographic and electronic structure with potential applications in numerous fields.^26,34^

### 2.2 Challenging chemistry of intermetallic compounds: an inorganic chemist's perspective

Forty years ago, H. Schäfer stated “Our knowledge of the number of compounds in the intermetallic systems, their chemical properties, and most importantly, the bonding mechanism in these phases are far less developed than in other chemical fields”.^35^ Today, we ask the question, what is the current understanding of these materials, compared to other inorganic compounds? The Materials Genome and Materials Project database^36–38^ contains a vast collection of inorganic extended solids of already known structure types (most of them include ionic solids, thermoelectric materials, and perovskites), which has accelerated the discovery of new materials, but the prediction of the chemical properties of intermetallic compounds may be more difficult than for any other class of inorganic solids. Difficulty arises because intermetallic compounds have a large variety of compositions, crystal structures, charge transfer phenomena, and chemical bonding scenarios.^26,34,39–41^ As a result of these complexities, they are rarely the focus of inorganic chemists, compared to other solids *e.g.*, oxides, halides, perovskites, and chalcogenides. Since several factors influence the chemistry of intermetallic compounds, only databases of known materials may not be helpful in the discovery of new compounds. One such factor is the assignment of the oxidation states of the constituent atoms in an intermetallic solid, which is not straightforward as in ionic and covalent solids, mostly due to partial charge transfer between constituent metals.^26^ Moreover, there is no specific electron counting scheme applicable to all classes of IMCs, making it a significantly more difficult task to comprehend this class of compounds compared to ionic solids. There have been some recent efforts to interconnect certain building blocks and polyhedra that occur in homologous intermetallic compounds and to determine the electronic stability of these phases by applying the 18-*n* rule.^42,43^ Predicting possible compositions that can exist in a particular system is also challenging. While many are likely able to rationalize the compositions of compounds such as KMnO_4_, HgI_2_, and Fe_3_O_4_, through simple oxidation state assignment and electron counting, it may not be straightforward for materials such as binary PtSb_2_, PtBi_2_, Pt_3_Co,^44^ PdSb_2_,^45^ Cu_5_Zn_8_,^19^ Pd_2_Zn_11_,^46^ Be_21_Pt_5_,^31^ and Sn_3_Sb_2_,;^47^ ternary ZrAl_2.6_Sn_0.4_,^48^ Ni_3_InSb,^49^ InPd_2_Cu,^50^ and many more. The situation becomes more complex for multinary systems like R_2_MoSi_2_C (R = Y, Gd)^51^ and Th_2_Au_5_Al_8_Si_2_.^52^

For non-intermetallic compounds, when there is a significant covalent character or partial ionic character in bonding, charge transfer occurs between bonding atoms, which can be explained easily in terms of periodic table trends, whereas analysis of the electronic structure of intermetallic compounds is complicated due to the presence of delocalized electrons. Nevertheless, electronic structure calculations and detailed chemical bonding analysis are often required to explain structural features and phase stability.^39,53^ It is important to recognize the experimental reality of synthesizing these intermetallic compounds, specifically, the growth of single-crystalline and phase-pure materials is often itself a challenge. For example, the presence of oxophilic metals (*e.g.*, Zn, Sn) as constituents may lead to oxide impurities along with the targeted intermetallic phase. If the targeted ternary intermetallic phase is a kinetic product, thermodynamically stable binary phases will interfere with the main phase, and in such an occasion, it is difficult to obtain phase-pure compounds suitable for measurements of physical and chemical properties.^26,34^

In solid-state chemistry, significant effort has been expended to develop structure–bonding–property relationships that connect fundamental variables like valence electron concentration (VEC, *e*/*a*), atomic size, and electronegativity. Due to the difficulties mentioned above, it is not a simple task to predict the crystal structure of intermetallic phases; even for the simplest equiatomic binary compounds, *e.g.*, PdZn,^54^ RhCd,^55,56^ RhZn.^57^ Recently, machine learning approaches have been used to predict the structures of AB-type RhCd,^55^ Heusler compounds,^58^ binary rare-earth intermetallics,^59^ Laves phases,^60,61^ and a few other classes of inorganic extended solids.^62^ Machine learning models are trained based on the datasets of experimentally known compositions and several other geometrical and electronic factors alongside the reported structure type.^63^

**Fig. 2:**
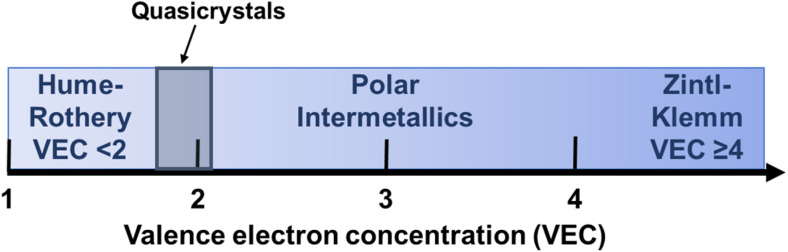
Trends of VEC for Hume-Rothery phases, quasicrystals (discussed later, grey zone), polar intermetallics, and Zintl–Klemm phases (left to right, respectively).

Since the standard electron counting rules (*e.g.* the 18-electron rule, the octet rule, Wade's rules) do not universally apply to all intermetallic phases, chemical bonding analysis has been employed to uncover commonalities and trends within different intermetallic structures.^39^ This analysis positions various compounds along a continuum between structures governed by the octet rule and localized two-center, two-electron bonds at one extreme (the most basic Zintl compounds, VEC ≥ 4) and perturbations on the free electron (Hume-Rothery phases, VEC < 2) at the other, with the polar intermetallic compounds emerging in the middle (Fig. 2).^53^

Our limited knowledge of the properties of intermetallic compounds suggests we have only scratched the surface of their potential for advancing chemical applications. New compounds are frequently discovered by the trial-and-error approach of synthetic investigations and more knowledge-based approaches, such as machine learning and extensive phase diagram explorations.^64^ Improving our ability to discover new IMCs through knowledge-based approaches is the key to tailoring IMC properties to the desired applications. This will only be possible when we can establish rigorous structure–bonding–property correlations.^26,39,53,65,66^

### 2.3 Three broad classifications of IMCs

Based on the valence electron concentration (VEC), intermetallic phases can be broadly classified into three major groups: Hume-Rothery phases, polar intermetallic phases and Zintl–Klemm phases (Fig. 2). Hume-Rothery phases mainly consist of transition metal elements and post-transition metal main group elements. The average valence electron per atom (*e*/*a*), also known as the valence electron concentration (VEC), plays an important role in determining the structure of Hume-Rothery phases.^24^ Theoretical investigations suggest over a range of VEC values, the Fermi surface interacts with the first Brillouin zone, which leads to the formation of a “pseudogap” at the Fermi level (*E*_F_) in the electronic density of states (DOS).^67,68^ The result is a decrease in the total kinetic energy of the valence electrons, stabilizing phases with appropriate crystal structures to maximize the interactions between the Fermi surface and the first Brillouin zone. For this reason, alloys with similar VEC values exhibit similar crystal structures, even when their constituent elements differ. Other factors, such as the radius ratio and atomic size, also play a significant role alongside VEC in determining the crystal structure of these phases.^68,69^

Zintl–Klemm phases are another class of intermetallic compounds consisting of electropositive (such as alkali and alkaline earth metals) and electronegative metals (such as p-block elements of group 13–15). In general, they occur at (*e*/*a*) ≥ 4. The large electronegativity difference between constituent elements leads to significant charge transfer between atoms, permitting the elements to obey the octet rule and behave like ionic salt-like compounds. The description of this class of compounds was developed by Zintl and later extended by Klemm.^70,71^ “This very simple idea, in my opinion, is the single most important theoretical concept (and how not very theoretical it is!) in solid-state chemistry of this century… it builds a bridge between solid-state chemistry and organic or main-group chemistry”, stated by Prof. Roald Hoffmann in 1987 regarding the charge transfer concept in Zintl–Klemm phases.^65^ NaTl (*cF*16, *Fd*3̄*m*, 227) was the first material that prompted Zintl's investigation into this class of intermetallic compounds.^70–72^

Polar intermetallic compounds fall between these two classes of compounds. They demonstrate partial charge transfer between their constituents (electropositive alkali, alkaline earth, or rare earth metals and more electronegative metals or metalloids), but complete valence electron transfer does not occur.^73^ Phase stabilization is conferred by the significant orbital overlap between the valence orbitals of the constituent elements. This class of compounds possesses both ionic and covalent interactions and, unlike Zintl phases, valence electron counting rules fail to justify the structure–composition relationship. Polar intermetallic compounds form a bridge between Hume-Rothery phases and Zintl phases.^74^

### 2.4 Other important classes of IMCs

#### 2.4.1 Laves phases

**Fig. 3:**
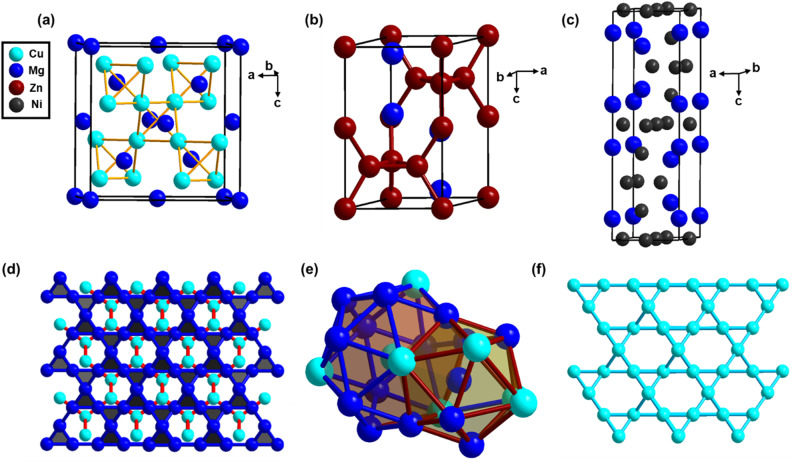
(top) Unit cells of (a) MgCu_2_, (b) MgZn_2_, and (c) MgNi_2_ Laves phases. (bottom) In the MgCu_2_-type Laves phase, a cubic diamond net of Mg atoms, along with Cu atoms, forms Cu_4_ tetrahedra sharing their vertices such that a pair of fused tetrahedra adopts a staggered conformation (d); 12-coordinated distorted icosahedra and 16-coordinated Frank–Kasper polyhedra: a tetracapped-truncated tetrahedron in the MgCu_2_-type Laves phase interpenetrating with each other (e); 3.6.3.6 Kagomé net formed by B (Cu) atoms (sky blue) in AB_2_ Laves phases (f).

The Laves phases are the largest group of intermetallic compounds with the general formula AB_2_, with more than 1500 representative A/B combinations.^75–77^ The basic three structure types are hexagonal MgZn_2_ (C14), hexagonal MgNi_2_ (C36), and cubic MgCu_2_ (C15) (Fig. 3a). Binary Laves phases with the general formula AB_2_ have an ideal radius ratio, *r*_A_/*r*_B_ = (3/2)^1/2^ ≈ 1.225, although the radius ratio values between 1.05 and 1.70 are often encountered experimentally.^78,79^ Along with geometric factors, electronic factors, such as the electronegativity differences between constituent elements A and B, direct the formation and stability of Laves phases.^75,80^ MgCu_2_ (C15) crystallizes in the *Fd*3̄*m* (227) space group in which Mg atoms form a cubic diamond net, while the Cu atoms form tetrahedral units. These tetrahedra share all the vertices in such a manner that each pair of fused tetrahedra adopts a staggered conformation (Fig. 3d). MgZn_2_ (C14) crystallizes in the hexagonal *P*6_3_/*mmc* (194) space group where the Mg atoms form a hexagonal diamond net, and the Zn atoms are again described as forming tetrahedral units. These tetrahedra alternately share their faces along the *c* direction, forming chains of trigonal bipyramids joined at their apex. MgNi_2_ (C36) also crystallizes in the hexagonal *P*6_3_/*mmc* (194) space group, where the Mg atoms form a hexagonal diamond net, and the nickel atoms form tetrahedral units, which are a combination of MgZn_2_ and MgCu_2_ type tetrahedral units. These structures can also be described by the stacking of 3.6.3.6 Kagomé nets and 3^6^ triangular nets. The stacking sequence differs in different structure types. The coordination polyhedra of the A/B atoms in various Laves phases are shown in Fig. 3e. The A atoms lie at the center of the tetra-capped truncated tetrahedra with a coordination number of 16 (12B + 4A), which is the so-called Friauf polyhedra. The B atoms lie at the center of the distorted icosahedra, which comprises six A and six B atoms (Fig. 3e). The tetrahedral intersections in Laves phases could be a potential site to efficiently locate hydrogen atoms with optimal host–guest interactions. These Laves phase hydrides significantly contribute to atomic hydrogen-based energy storage.^81^

#### 2.4.2 Heusler compounds

**Fig. 4:**
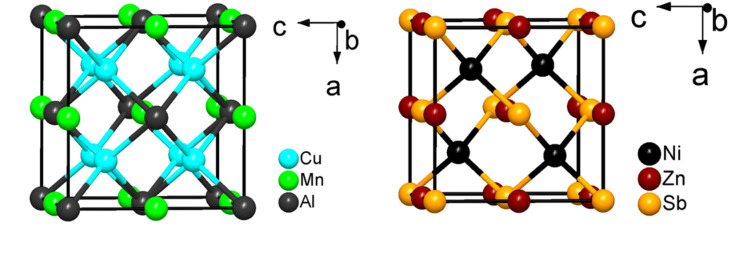
Unit cell of a full Heusler compound (left, Cu_2_MnAl) and a half-Heusler compound (right, NiZnSb).

Heusler compounds, named after the German physicist Fritz Heusler, who first studied them in the early 20th century, constitute a class of ternary intermetallics.^82^ There are two structure types in the Heusler class of compounds; full Heusler, X_2_YZ (XX′YZ), with the L2_1_ structure type (*Fm*3̄*m*, Cu_2_MnAl-type) and half Heusler, XYZ, with the C1_b_ structure type (*F*4̄3*m*, MgAgAs-type), where X and Y are transition or rare-earth metals and Z is a main-group element (Fig. 4). The structure of full Heusler compounds can be described by four interpenetrating fcc sublattices of the constituent elements. The removal of X or X′ leads to half-Heusler structures.^83^ Tetragonally distorted, hexagonal and quaternary variants are also known in this class of intermetallic compounds.^82,83^ Although Heusler and half-Heusler phases have long been recognized as magnetic materials, they can also be heavy fermion systems and superconductors if rare-earth elements are included.^84,85^ Their potential in heterogeneous catalysis has been investigated more recently.^86–89^

#### 2.4.3 The γ-brass phases

**Fig. 5:**
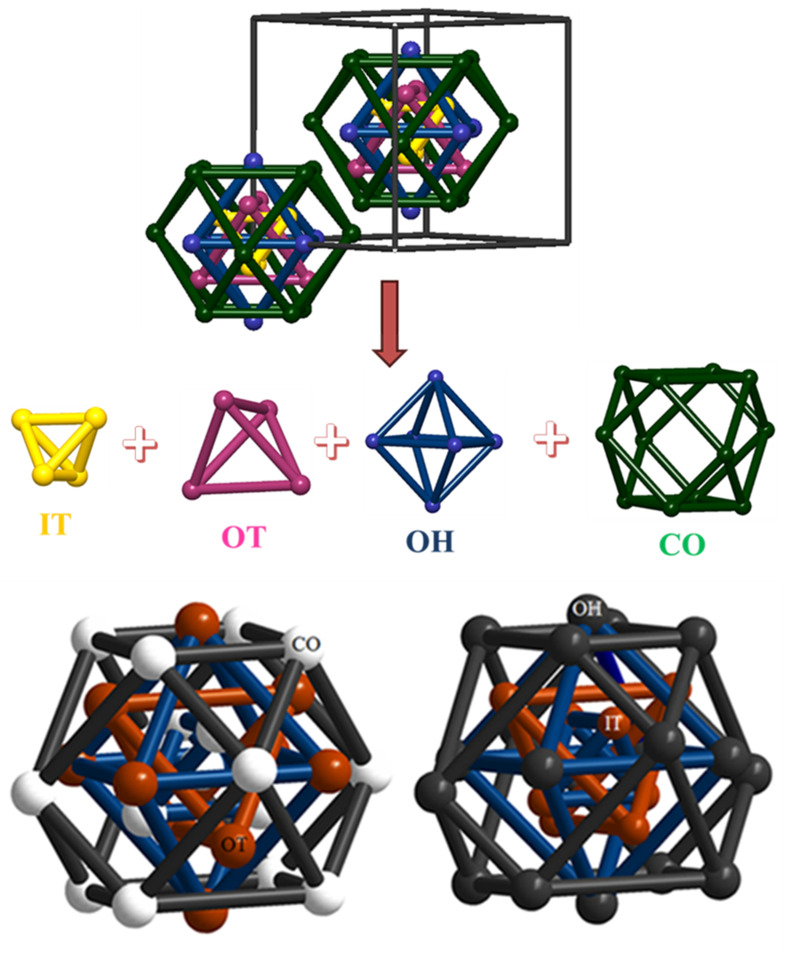
(top) A 26-atom γ-brass cluster shown in the unit cell of typical gamma-brass; IT (yellow), OT (pink), OH (blue), and CO (green). (bottom) Comparison between the atomic distributions of two 26-atom γ-clusters, Cu_5−*x*_Zn_8+*x*_ (left)^19^ and Cu_4_Cd_9_ (right):^91^ brown spheres: Cu atoms; white spheres: Zn atoms; and black sphere: Cd atoms.

γ-Brass Hume-Rothery phases, the archetype of Cu_5_Zn_8_, captivate researchers' attention for their structural complexity and intricate phase relations.^19^ According to the Hume-Rothery rules, the γ-brass-type phases generally occur with a VEC of ∼21/13.^90^ The electronic structure calculations of the γ-brass-type compounds demonstrate the existence of a pseudo-gap at the Fermi level. This is caused by the interaction between the Fermi surface and the Brillouin–Jones zone boundary defined by the {411} and {330} Bragg planes. This pseudo-gap opening lowers the electronic energy and provides structure stabilization. The ideal γ-brass-type structures crystallize in space group *I*4̄3*m* (217) with a lattice parameter, *a* ≈ 9 Å, and contains ∼52 atoms per unit cell. The γ-brass type structures can be described by a 26-atom cluster formed by four atomic shells. Moving outward from the center of the cluster, these shells can be described as follows: four atoms forming an inner tetrahedron (IT), an outer tetrahedron (OT) of four atoms sitting above the faces of the IT, six atoms forming an octahedron (OH) such that each OH atom lies above the edge of the OT, and a distorted cuboctahedron (CO) of twelve atoms placed above the edges of the OH. In γ-brass Cu_5_Zn_8_, Zn atoms occupied the IT and CO sites, whereas Cu atoms occupied the OT and OH sites (Fig. 5). Electronic structure calculations based on Mulliken population analyses demonstrated the less electronegative element in a γ-brass variant prefers the IT and CO sites, whereas the more electronegative element prefers the OT and OH sites.^19^ It is noteworthy that the atomic distributions in the homologous compound Cu_4_Cd_9_ are different from those of Cu_5_Zn_8_, where the difference between the atomic radius of Cu and Cd and the chemical bonding scenario play a decisive role (Fig. 5).^91^

#### 2.4.4 Complex intermetallic compounds

Complex intermetallic compounds^34,92^ (often called complex metallic alloys, CMAs) form periodic structures with numerous unit cells containing several hundred to several thousand atoms in their unit cell. They represent a subclass of intermetallic phases and have attracted attention in the past few decades owing to their structural complexity, solid solution behavior, stabilization mechanisms, and diverse physical properties. Many of them fall in the category of quasicrystals (solids with a long-range order and quasiperiodic atomic structures) and the approximant phases of quasicrystals (series of periodic structures converging to quasicrystals).^93^

One of the first examples of a complex IMC is NaCd_2_. Despite its simple stoichiometry, the diffraction patterns were found to be so complex that solving the structure was nearly impossible 100 years ago. It took more than 30 years before the structure was successfully solved; it crystallized in the high symmetry *Fd*3̄*m* (227) space group with a unit cell of ∼30 Å that contains 1152 atoms.^94^ Our ability to accurately determine the crystal structure of complex intermetallic compounds has significantly improved, especially with the development of electron diffraction. Nevertheless, the complexity of these structures challenges our ability to understand their formation.

The bulk electronic structures of many CMAs feature real or pseudogaps in the density of states at the Fermi level. The electronic stability of CMAs is often associated with a definite valence electron concentration (*e*/*a*), which is attributed to the previously mentioned Hume-Rothery mechanism.^25^ The complex bulk crystal structures of CMAs mean they often possess rather irregular and strongly corrugated surfaces, which leads to difficulties in the determination of the atomistic details of the surface. To deal with the complexity of these surfaces, a combination of STM and *ab initio* DFT calculations was used to unveil both the energetics and electronic and geometric structure of the stable surfaces of CMAs.^95^

Intermetallic carbides^96^ and hydrides,^97^ though less extensively studied than their binary and ternary counterparts, represent important subclasses of IMCs that exhibit unique structural motifs and offer intriguing properties related to heterogeneous catalysis and atomic hydrogen storage.

#### 2.4.5 High entropy alloys (HEA) and high entropy intermetallics (HEI)

**Fig. 6:**
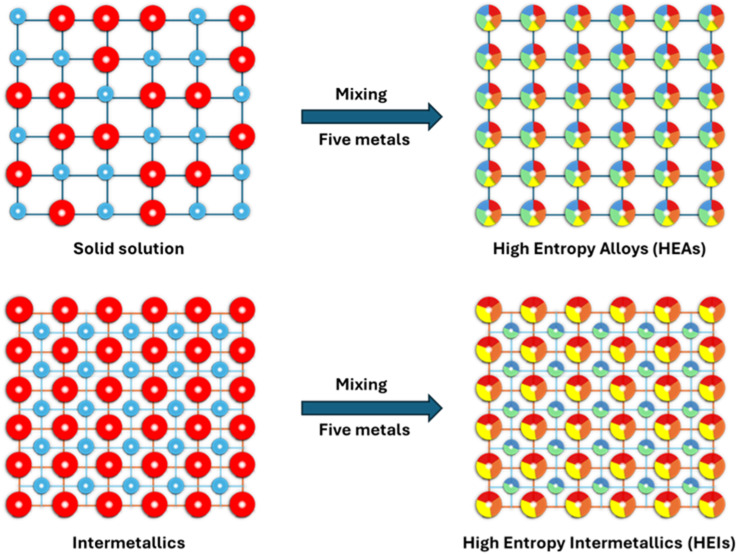
Schematic of atomic distribution in quinary high-entropy alloys and high-entropy intermetallics (HEIs).

The term “HEIs” was first coined by Tsai in 2016 to define a class of intermetallic compounds composed of multiple principal elements.^98^ Mixing at least five (or more) metals with equiatomic or nearly equiatomic ratios leads to the formation of so-called high entropy materials.^98–100^ Materials with a random distribution of all metals are referred to as high entropy alloys (HEAs), whereas materials in which metals have site-specific occupancies are known as high entropy intermetallics (HEIs) (Fig. 6). HEAs lack long-range chemical ordering owing to the random site occupancy of a given lattice site by different elements. Furthermore, the formation of HEAs is entropy-driven because of the large number of constituent elements exhibiting mixing effects, which enhance their thermodynamic stability.^101^ On the other hand, HEIs exhibit long-range atomic ordering because each lattice site is limited to a certain subset of elements. Retaining an ordered structure despite the large number of constituent elements implies HEIs are thermodynamically stable because of enthalpic stabilization.^102^

### 2.5 Coloring problem in intermetallic compounds: site preference in the bulk crystal structure

In solid-state chemistry, the ‘coloring problem’^66^ refers to the difficulties associated with determining the distribution of different atoms within a given crystal structure. The connectivity and arrangement of different atoms in extended solids strongly affect their physical and chemical properties. The fundamental question is “Given a molecular or extended network and several different types of atoms or chemical groups, in what arrangement is the energy of the system minimized for a fixed stoichiometry?” In other words, which ‘coloring scheme’ is favorable over another? It is also important to consider why one ‘coloring scheme’ is favorable over another. Determining the preferred ‘coloring scheme’ is key to establishing structure–property relationships in catalysis and other research areas.^103^

The accurate assignment of constituent atoms among different sites is primarily investigated by X-ray diffraction (XRD). The assignment of atoms from XRD may not be accurately determined for elements that neighbor each other on the periodic table because they have similar X-ray scattering factors and therefore are indistinguishable using XRD. In these cases, neutron diffraction is usually employed to differentiate the neighboring elements under investigation. However, some elements commonly used in solid-state materials are neutron absorbers (*e.g.*, Cd, B, Gd), which rules out the application of neutron diffraction to characterize intermetallics containing these elements or require the use of expensive isotopes. Additionally, neutron diffraction typically requires a beamline neutron source, complicating the accessibility of the measurement. Due to experimental challenges, the site distribution of atoms in structures is often addressed using first-principles calculations.

Density Functional Theory (DFT)-based^104^ total energy calculations are executed on various models to accurately determine the coloring scheme preferred by the constituent elements in a given structure. The factors influencing one coloring scheme over others are addressed by the energetic contributions from site potentials and pairwise interatomic potentials. Valence electron population analysis (Mulliken or Löwdin's population analysis)^105,106^ at each site of all configurations is often required to accurately determine the preferred coloring scheme. For site preference, site energy is an important contributing factor. The more electronegative atoms usually prefer sites with a higher electron population to lower the electronic energy of the system (topological charge stabilization).^107,108^ Conversely, bond energy factors can also contribute to the observed site distribution pattern in the same structure, which can be addressed by comparing the number of homoatomic and heteroatomic contacts in all possible coloring schemes. If a greater number of heteroatomic contacts lead to a lower total energy, the correct coloring scheme will likely contain a higher number of heteroatomic contacts. In many intermetallic structures, both the site energy and bond energy factors contribute to the observed distribution pattern of the constituents.

### 2.6 Relationship between crystal structure, electronic structure, partial charge transfer, and core chemical bonding scenario in the IMCs

To understand the electronic stability of any intermetallic compound from a chemical standpoint and specific site substitution patterns, electronic structure calculations were performed. The electronic density of states (DOS) uncovers ‘where the electrons are’ and provides an overall idea about the electronic environment of intermetallic compounds. The crystal orbital Hamilton population (COHP)^109^ method is a theoretical bond analysis tool for solids that ‘partitions the band-structure energy into orbital pair interactions’ (orbital-space bonding description). From a chemical point of view, COHP is a “bond-weighted density-of-states between two adjacent atoms within their interacting distance”.^110^ A COHP diagram is generally plotted alongside the DOS, which indicates the bonding and anti-bonding contributions to the band-structure energy. DOS and COHP provide a clear picture of the complete electronic structure of the material (*i.e.*, where the electrons are and how they bond to each other). The integrated electronic DOS yields the total number of electrons in the system under investigation, whereas the integrated COHP (ICOHP) provides information regarding the bond strength (in eV or kJ mol^−1^). After the calculation of the self-consistent electronic wave function (SCF), both the DOS and COHP can be determined, allowing important chemical information to be extracted.^111,112^ Another method to quantify covalent bonding in solids, crystal orbital bond index (COBI), has been introduced recently.^113^ COBI is very similar to COHP and relates directly to the ‘bond order’. Additionally, COBI can identify multicenter interactions in periodic extended solids.^53,113^ TB-LMTO (tight binding linear muffin-tin orbital)^114^ developed by Andersen *et al.* and LOBSTER (local-orbital basis suite toward electronic structure reconstruction)^110,115^ developed by Dronskowski *et al.* are the two widely used open-source tools to calculate COHP in periodic solids. The latter method uses plane-wave basis sets and allows the extraction of a chemically useful projected COHP (pCOHP).

**Fig. 7:**
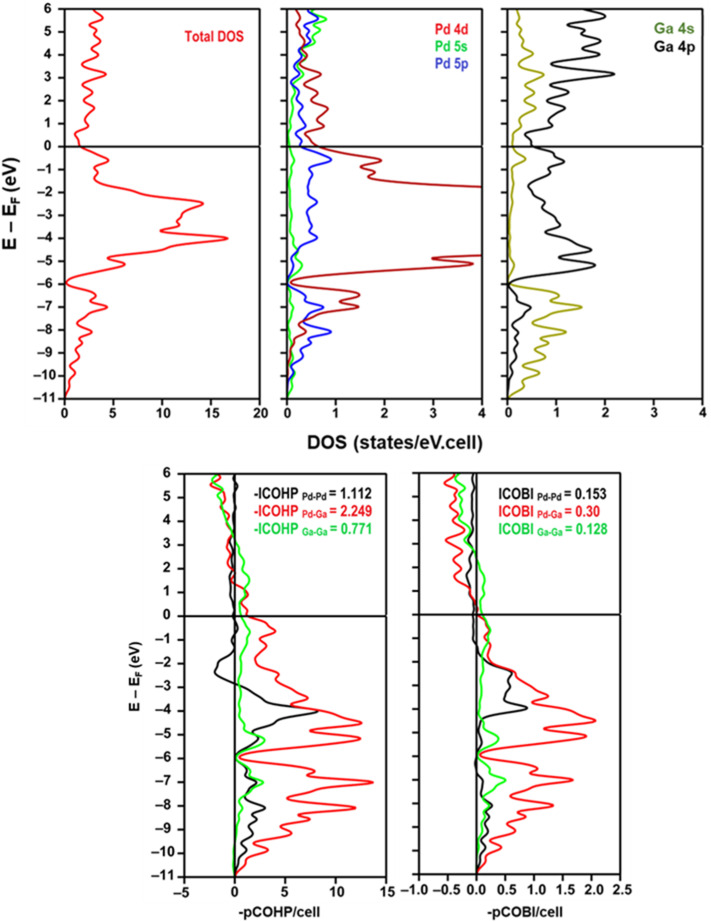
(top) Electronic density of states of the catalytically relevant intermetallic compound PdGa.^27^ (bottom) COHP and COBI plots of individual interactions in the structure of PdGa (with average integrated values). Adapted from ref. 27 and calculated by LOBSTER^115^ for illustrative purposes. Structural optimization was performed using Quantum Espresso,^116^ and plots were created using wxDragon.^117^

For illustrative purposes, we calculated the DOS, COHP and COBI of a catalytically relevant intermetallic compound PdGa (FeSi-type, *P*2_1_3) (Fig. 7) using the crystal structure from ref. 118. The Fermi level of the density of states is significantly different from that of a pure metal with a hint of a pseudogap, and the overlap between the Pd 4d and Ga 4s, 4p states leads to strong and partially covalent Pd–Ga heteroatomic interactions. Among the three possible interactions in the structure of PdGa, the average integrated COHP (and COBI) values were highest for the Pd–Ga bonds, which contributed to the structural stability of PdGa.

In the context of heterogeneous catalysis, these calculations extract the chemical bonding interactions, bond strengths, and structural stability of the catalyst,^119,120^ but can also reveal information about substrate–catalyst surface interactions and may provide insightful mechanistic details by revealing the different possible adsorption modes of a substrate on the surface of a catalyst.^121^

The Quantum Theory of Atoms in Molecules (QTAIM),^122^ developed by Richard Bader, is another intuitive method for breaking molecules down into atoms to understand their mutual interactions. The definition of an atom is based only on the density of charge and divides atoms using a technique known as zero flux surfaces.^123^ A definition of chemical bonding with numerical values for bond strength can also be provided by this theory. Henkelman's group developed a grid-based approach that can be successfully applied to extended solids, *e.g.*, intermetallic compounds. This approach allows for the analysis of the large grids generated from plane wave-based density functional theory calculations.^124^ A similar approach is implemented in the novel functional ‘electron localizability indicator’ (ELI), which has been proven useful in investigating the core chemical bonding situation in periodic solids. ELI can be broken down into partial orbital contributions (unlike the classic ‘electron localization function’,^125^ ELF).^126^ A combination of the electron density (ED) and electron-localizability indicator (ELI-D) can reveal details about a larger range of interactions, from single pairs to multi-center bonds. Both the distinct core regions and the shared valence region can be identified in the ELI-D distribution. The distribution is spherically symmetric for non-interacting atoms, and the existence of atomic interactions is indicated by any divergence from the spherical distribution. Local ELI-D maxima (attractors) in the valence region help visualize bonds in real space.^127^

**Fig. 8:**
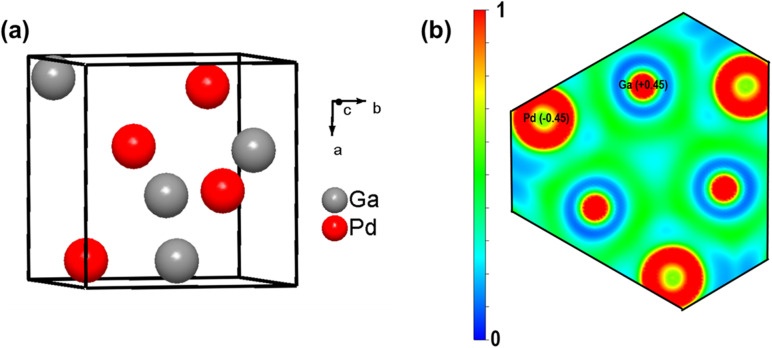
(a) Unit cell of FeSi-type PdGa. (b) ELF plot on a (1̄ 1̄ 1̄) slice of PdGa [adapted from ref. 27 and 118]. The average Mulliken charge on the Pd site is −0.45 and on Ga site is +0.45, indicating partial charge transfer from Ga to Pd. The ELF was calculated using Quantum Espresso,^116^ and the plot was drawn using VESTA.

Unlike Bader's QTAIM,^122^ Mulliken^106^ and Löwdin^105^ population analyses are based on the electron density matrix, using a suitable and balanced basis set of atomic-like orbitals and considering the overlap population of the orbitals. Despite being a simplified approach, Mulliken populations are still widely used to determine the atomic charges of the constituent elements of non-molecular solids. For example, the ELF plot (Fig. 8) on the (1̄ 1̄ 1̄) slice of cubic FeSi-type PdGa shows a significant electron density between Pd and Ga (partial covalent character), and Mulliken charges on Pd and Ga indicate the direction of charge transfer from Ga to Pd, which correctly aligns with the expected trend based on their electronegativity.^27,118^ Wider green regions between the Ga atoms indicate more profound covalency of Ga–Ga interactions. The implications of the electronic charge transfer, strong Pd–Ga heteroatomic bonds of intermetallic PdGa on its performance in the selective hydrogenation of acetylene are discussed in Section 3.4.

The contribution of the atomic sizes of the constituent elements in an intermetallic structure remains vague in the abovementioned experimental and theoretical approaches. An intuitive and relatively newer approach, called density functional theory-chemical pressure (DFT-CP) analysis, introduced by Fredrickson *et al.*^128^ considers the influence of atomic size as the ‘most pronounced factor’ while taking into account chemical interactions between the constituents. The tools developed by Fredrickson *et al.*^129^ can visualize the local chemical pressures on individual elements inside the unit cell of a periodic structure. This aids in understanding whether bond formation is inhibited by steric repulsion or by insufficient electronic support in homologous compounds. This method has been applied to relatively simple structure types to rather complicated structures exemplified by complex metallic alloys and quasicrystal approximants.^130,131^

Our understanding of the formation, structural stability, site preference of constituent elements, and differences in the structure types of homologous series of compounds in intermetallic solids remains incomplete despite the varied approaches used to characterize their electronic structure chemical bonding. As discussed above, this includes quantum theory of atoms in molecules (QTAIM),^122^ electron localization function^125^ (and electron localizability indicator),^126^ orbital space chemical bonding analysis (*e.g.*, COHP,^109,112^ COBI^113^), DFT-CP analysis (chemical pressure).^128^ A single approach often fails to explain the intricate structural phenomenon in intermetallic solids. For example, the unprecedented site preference of Zn in the structure of ordered tetragonal Cu_6_Zn_2_Sb_2_ can be explained based on Mulliken's and Lowdin's charge population analysis (topological charge stabilization), but Bader's QTAIM failed to arrive at the same conclusion.^132,133^ A recent study of the CoSn-type intermetallic compound (*P*6/*mmm*) NiIn_1−*x*_Sn_*x*_ (*x* = 0.7–0.9)^134^ has shown the specific site-preference of Sn can be explained by Bader's charge, Mulliken's and Lowdin's charge population and real space charge density analyses. All these calculations led to the same conclusion (unlike the previously mentioned Cu_6_Zn_2_Sb_2_)^133^ regarding the experimentally established (based on X-ray and neutron diffraction) ‘coloring scheme’. However, one should be extremely careful regarding the choice of basis-sets (orbitals) in orbital-space calculations.

These different approaches can lead to significant differences in the perception of chemical bonding in extended inorganic solids. For example, Bader's density-based approach identifies a new bonding mechanism, *i.e.*, metavalent bonding/electron-deficient bonds in phase change memory materials,^135^ topological insulators, and halide perovskites. On the other hand, the orbital-based approach finds this bonding scheme “unjustified” and identifies the bonding in these electron-rich materials, 3c–4e (‘hypervalent’); this approach believes ‘electron density’ alone is insufficient and requires analysis of the electronic wave functions (the orbitals).^136^

### 2.7 Knowledge of the bulk is not enough: importance of surface structure and composition in heterogeneous catalysis

The previous section discussed the structure(s) of different type(s) of intermetallic compounds and the fundamentals associated with them. However, when considering these materials from the perspective of heterogeneous catalysis, the surface and electronic structure are more important because they determine the nature of the active sites. From a heterogeneous catalysis perspective, site preferences (the ‘coloring scheme’ on the surface) and the most stable surface facets (under the influence of adsorbates) are important because they determine the local environment of the exposed active sites. Site preferences in the bulk can be found by completing bulk energy calculations for different possible configurations within the experimentally determined model structure; however, this does not provide information about surface site preferences. Therefore, surface energy calculations are needed to provide accurate predictions of the distribution of active and inactive constituent elements on the surface.^95^

**Fig. 9:**
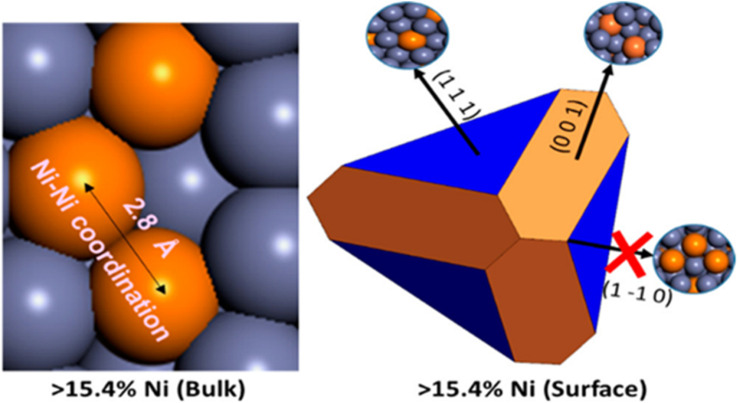
(left) Ni–Ni–Ni trimer present as determined by experiments in the bulk of Ni_9_Zn_43_. (right) The corresponding Wulff construction showing that the Ni–Ni–Ni trimer containing {1 1̄ 0} facet is not energetically favored to be exposed. The orange and grey spheres represent Ni and Zn, respectively. Reprinted with permission from ref. 137, copyright 2017, the American Chemical Society.

The importance of surface structure is highlighted with Ni–Zn γ-brass compounds that contain different Ni atomic percentages (Ni_8_Zn_44_, Ni_9_Zn_43_, Ni_10_Zn_42_, Ni_11_Zn_41_).^137^ While it was anticipated the catalyst would become more active with increasing Ni content, the rate of H–D exchange was invariant with the bulk composition. Neutron powder diffraction, in combination with bulk energy calculations, was used to investigate the bulk structure of the compounds, including site preferences, and additional Ni in the higher Ni content preferred octahedral sites (OH). Thus, the isolation of the Ni-sites was lost, and the bridging Ni atoms in the OH sites formed Ni–Ni–Ni trimers. The results of the bulk structure investigation do not provide any explanation for the insensitivity of the rate of H–D formation to Ni content, requiring consideration of the most stable surface facet(s) and the extent of Ni exposure on those facets. Surface energy calculations demonstrated that surface facets containing Ni–Ni–Ni trimer sites were energetically unfavorable and therefore not exposed (Fig. 9). Since the additional Ni in these compounds remains sub-surface, it has no measurable impact on catalysis.^137^

The homologous γ-brass compounds in the Pd–Zn system [Pd_8_Zn_44_, Pd_9_Zn_43_, Pd_8_MZn_44_ (M = Cu/Ag/Au)] differ significantly from each other (and from the Ni–Zn system) in terms of their calculated surface composition and surface ensembles. A combined experimental and theoretical effort has revealed that M (Cu/Ag/Au) has a strong substitution preference on the Zn atoms of the OH-site, whereas Pd exclusively occupies the OT-site.^138^ Surface energy calculations led to the conclusion that, different catalytic ensembles on the surface, such as monomer Pd_1_ (*i.e.*, Pd–Zn–Pd) for Pd_8_Zn_44_, and trimer Pd_3_ (*i.e.*, Pd–Pd–Pd) sites for Pd_9_Zn_43_, are responsible for at least six orders of magnitude difference in the rates of ethylene hydrogenation.^139^ A very specific site occupancy pattern of Pd and Zn in the Pd–Zn γ-brass IMCs lead to the transition from Pd_1_ monomer active sites in Pd_8_Zn_44_ (15.4 at% Pd) to Pd_3_ sites in Pd_9_Zn_43_ (17.3 at% Pd). When a third element was introduced with the nominal composition Pd_8_MZn_44_ (M = Cu/Ag/Au), the coinage metal showed a strong preference for the OH-site which modifies the catalytic active site ensemble to Pd–M–Pd (Fig. 11).^138,140^ These modifications have influenced the performance of these intermetallic catalysts for the selective hydrogenation of acetylene, and these aspects are discussed in Section 3.2.

Alongside the DFT-based energy calculations, experimental surface characterization techniques are extremely important to establish a complete understanding of the structure (surface)–catalytic property relationship of IMCs. A detailed surface characterization of PdGa (FeSi-type) by Kovnir *et al.*^141^ compared the bulk structure of PdGa with its surface structure. *In situ* XRD and *in situ* EXAFS (Extended X-ray Absorption Fine Structure) proved the structural stability of PdGa during the selective hydrogenation of acetylene. XPS (X-ray photoelectron spectroscopy) measurements confirmed the electronic structure of the surface closely resembled the bulk. Comparative CO adsorption experiments (Fourier transform infrared spectroscopic measurements, CO-FTIR) of PdGa and a commercially available reference catalyst, Pd/Al_2_O_3_, revealed the isolated nature of the active Pd sites on the surface of PdGa.^141^ The aspects of this catalyst related to the selective hydrogenation of acetylene are discussed in Section 3.3.

## 3. Part II: recent investigations of intermetallic compounds as selective hydrogenation catalysts

### 3.1 Alloying introduces limited flexibility in active site composition

Noble metals such as rhodium, ruthenium, palladium, and platinum are frequently employed as heterogeneous catalysts for various hydrogenation reactions because of their high activity. However, these metals are expensive, and their high activity is typically achieved at the expense of poor selectivity. Deactivation of these expensive materials *via* coking imposes the need for catalyst regeneration or replacement.^142^ The inherent lack of flexibility in the active site composition of monometallic catalysts implies a reliance on the intrinsic properties of the metal surface, which rarely results in the optimum adsorbate binding energy, as described by the Sabatier principle.^143^

Alloying these noble metals, typically with a less catalytically active metal, offers the ability to modify the active sites and, as such, the potential to improve catalytic properties, particularly selectivity and stability. For instance, Ag_*x*_Pd_1−*x*_ alloys have been employed industrially for many years in the selective hydrogenation of acetylene to ethylene due to a higher selectivity compared to pure Pd.^144–147^ X-ray absorption spectroscopy studies, in combination with classic electronic structure calculations, have demonstrated the selectivity improvement is largely due to an electronic effect; charge transfer from Ag to Pd results in an increase in filled Pd 4d-states.^148–150^ The increased electron density in the Pd 4d-states results in weaker binding energies of unsaturated hydrocarbons (ethylene and acetylene) since they are electronically rich and typically donate electron density to Pd. Although this adsorption behavior may lead to decreased rates of acetylene hydrogenation, the weakened ethylene adsorption decreases the propensity for over-hydrogenation and therefore improves selectivity.

The presence of electronic modifications does not eliminate the possibility of structural changes to the surface of the active metal, which may also contribute to improved catalyst performance. Infrared studies using CO as a probe molecule demonstrated an increase in the isolation of Pd sites upon alloying with Ag correlated with improved selectivity in acetylene hydrogenation.^151^ It was proposed that an increase in the isolation of Pd sites may result in a decrease in hydrogen concentration around the active site, and therefore allow ethylene to desorb before over-hydrogenation. This highlights one of the key challenges in understanding the catalytic behavior of these materials: distinguishing the contribution of electronic and geometric effects on catalytic performance. The origin of the increased selectivity over Ag–Pd alloys is further complicated because these alloys are randomly substituted (see substitutional alloys in Section 2.1 above), implying that several possible active site configurations contribute to the observed catalytic behavior. Additionally, the lack of any significant preference for a particular lattice position by the constituent elements means catalyst pre-treatment (*e.g.*, calcination, reduction) or changes in the process conditions may result in significant restructuring of the catalyst.^147,151–153^

Nørskov and Studt *et al.*^154,155^ established scaling relationships between acetylene and ethylene adsorption energies on transition metal surfaces as a function of the adsorption energy of the methyl group. Acetylene and ethylene form four and two σ-bonds, respectively, with the surface of the transition metal and these adsorption energies scale with respect to the adsorption energy of a methyl group. A catalyst that weakly binds a methyl group, compared with pure Pd, will be more selective than pure Pd. Their prediction identified several alloys potentially active and selective catalysts, *e.g.*, Pd–Au, Pd–Ag, Pd–Ga, and Pd–Sb.^155^ The same work identified alloys of Ni, a cheaper alternative to Pd, as selective catalysts. The results were experimentally verified for Ni–Zn alloys, and it was found the alloy with the highest Zn content had a higher selectivity to ethylene than the widely used Pd–Ag alloys.^154^

**Fig. 10:**
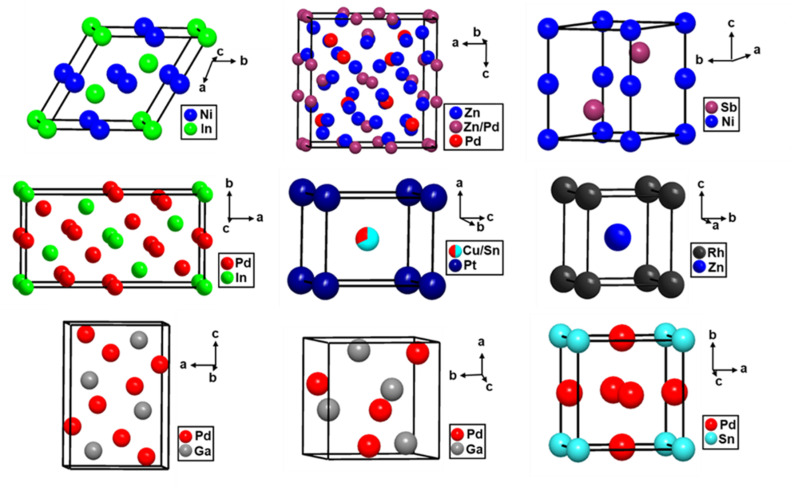
Structures of a few intermetallic compounds relevant in alkyne hydrogenation: CoSn-type NiIn, γ-brass type Pd_9_Zn_43_, NiAs-type NiSb (top left to right); Rh_5_Ge_3_-type Pd_5_In_3_, CuAu-type PtCu_0.67_Sn_0.33_, CsCl-type RhZn (middle left to right); Co_2_Si-type Pd_2_Ga, FeSi-type PdGa, and Cu_3_Au-type Pd_3_Sn (bottom left to right).

Intermetallic compounds offer the opportunity for significantly greater control over the electronic and geometric structure of active sites, largely due to specific site preferences in the crystal structure, which allows for the development of rationally designed catalysts for selective hydrogenation. This strong site preference also has implications regarding stability since it can mitigate the issue of segregation under pre-treatment and/or reaction conditions, which is often a challenge with random alloys. The complex bonding and electronic structure of intermetallic compounds suggest they can have very different catalytic properties compared to the parent metals, which allows for the potential to develop active and selective catalysts with cheaper and more abundant elements. There is an increasing interest in the use of intermetallic compounds as heterogeneous catalysts; the next section of this review focuses on their use as model catalysts in selective alkyne hydrogenation reactions. The crystal structures of a few catalytically relevant intermetallic compounds are depicted in Fig. 10.

### 3.2 Controlling active site composition and configuration using intermetallic compounds for the rational design of catalysts for selective hydrogenation reactions

**Fig. 11:**
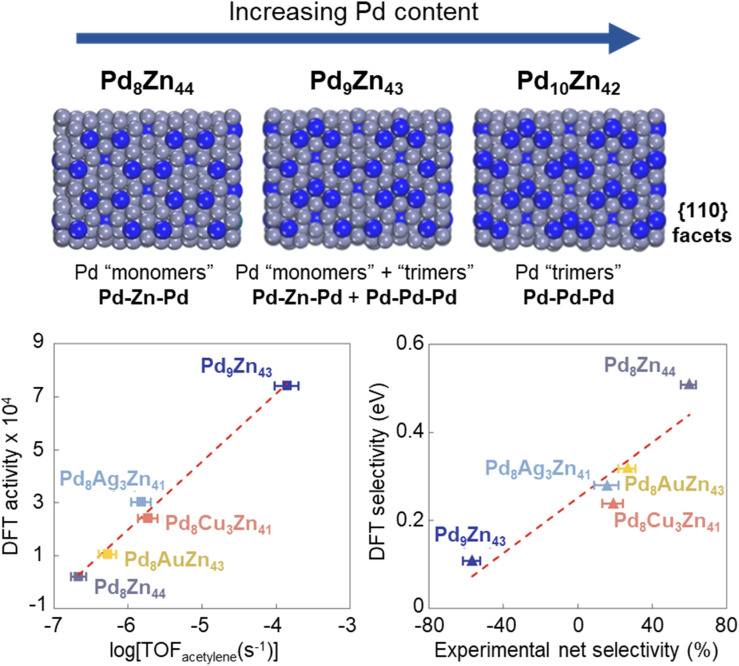
(top) Visualization of Pd–Zn–Pd, Pd–Pd–Pd, and Pd–M–Pd (M = Cu/Ag/Au) trimers in the {110} facet of Pd–Zn γ-brass intermetallic compounds.^140^ (bottom) The activity (left) and selectivity (middle) show strong agreement between experimental kinetics and DFT. Reprinted with permission from ref. 140 copyright 2022, Nature.

The specific site preferences found in intermetallic compounds allow precise control and accurate prediction of the composition and configuration of active sites present on the surface of the catalyst. The superior control offered by intermetallic compounds was exemplified in a recent study that explored Pd–Zn γ-brass intermetallics for the selective hydrogenation of acetylene to ethylene.^140^ The lowest energy facet, {110}, exposes the OT–OH–OT ensemble (see Section 2.7 above), with the composition of this ensemble being highly dependent on the stoichiometry of the intermetallic compound. For instance, in the case of Pd_8_Zn_44_, the ensemble consists of Pd–Zn–Pd resulting in Pd sites completely isolated by Zn, denoted as Pd “monomers”. Subtle increases in the stoichiometric Pd content lead to the introduction of Pd–Pd–Pd ensembles, denoted as Pd “trimers”, while Pd_9_Zn_43_ consisting of a mix of Pd trimers and monomers and Pd_10_Zn_42_ consisting of only Pd trimers (Fig. 11). Pd_8_Zn_44_, composed of completely isolated Pd monomer sites (Pd–Zn–Pd), was highly selective for ethylene but with a low rate of acetylene hydrogenation. The Pd_3_-trimer sites (Pd–Pd–Pd) on Pd_9_Zn_43_ were found to be significantly more active for the hydrogenation of acetylene; however, they were also at least ∼10^6^ times more active for ethylene hydrogenation, resulting in a significant decrease in selectivity. The di-σ-binding of acetylene across a Pd monomer “pair” permits H co-adsorption followed by C≡C hydrogenation. However, π-bound ethylene prevents H_2_ dissociative adsorption, H co-adsorption, and C=C hydrogenation at the isolated Pd-sites. The nuclearity of the active sites (*i.e.*, Pd monomers *versus* trimers) is directly correlated to both the activity and selectivity of the catalyst during the selective hydrogenation of acetylene.

Further modification of the active site was also found to be possible through the inclusion of small amounts of a coinage metal to produce intermetallic compounds with a composition of Pd_8_MZn_43_ (M = Cu, Ag, Au). With this stoichiometry, Pd was found to preferentially occupy the OT sites and the coinage metal occupied OH sites,^138^ resulting in Pd–M–Pd ensembles. The activity and selectivity of the Pd–M–Pd ensembles were intermediate between the Pd monomers and the Pd trimers in the binary system. Therefore, both the nuclearity and composition of the active site ensemble were found to determine the overall catalyst activity and selectivity in acetylene hydrogenation, which can be controlled with the careful and rational design of intermetallic compounds.^140^

The configuration of the active site was also found to be crucial to achieve high selectivity in acetylene hydrogenation with Pd–In intermetallic compounds.^156^ PdIn (*Pm*3̄*m*) has isolated Pd sites on the lowest energy {110} surface, however Pd_3_In (*P*4/*mmm*) has Pd_3_-trimer sites on the lowest energy {111} surface. These intermetallic catalysts displayed notably different selectivity in acetylene hydrogenation; PdIn was found to have a selectivity of 92%, whereas Pd_3_In had a selectivity of only 21%. DFT calculations for PdIn showed the desorption energy for ethylene was lower than the hydrogenation barrier because of the isolation of the Pd sites by In. However, for Pd_3_In, the ethylene desorption energy was similar to the hydrogenation barrier, consistent with the increased rate of ethylene hydrogenation and poor ethylene selectivity. The distance between adjacent Pd atoms was therefore identified as a structural descriptor of ethylene formation during the selective hydrogenation of acetylene.^156^

Modification of the active site configuration in intermetallic compounds has implications not only for selectivity but also stability. Spanjers *et al.*^157^ explored the effect of the Zn content in Ni-based intermetallics on the active site geometry for the selective hydrogenation of acetylene. Monometallic Ni catalysts are prone to oligomerization during acetylene hydrogenation, which leads to the formation of by-products which contribute to catalyst deactivation.^158^ Ni–Zn intermetallic catalysts were proposed to significantly reduce the formation of these oligomeric species, which should limit catalyst deactivation and improve the selectivity toward ethylene.^157^ Three intermetallic systems were chosen in this study: cubic Ni_4_Zn (fcc-Cu type, with 2.4 Ni neighbors replaced by Zn), tetragonal NiZn (AuCu-type, with 8 Zn nearest neighbors) and Ni_5_Zn_21_ (gamma-brass type, with the Ni sites completely isolated by Zn). The amount of observed oligomerization during acetylene hydrogenation was found to decrease with increasing Zn content (and increasing Ni isolation); however, DFT calculations initially appeared to contradict this result since the barrier for C–C bond formation was found to be lower for NiZn compared to monometallic Ni. The inclusion of Zn was also found to lower the acetylene binding energy, and the authors demonstrated through the implementation of a Langmuir–Hinshelwood model that the lower acetylene binding energy would result in significantly lower acetylene surface coverage. Since oligomerization occurs through the reaction of an adsorbed acetylene molecule with a partially hydrogenated acetylene intermediate, the lower surface coverage of acetylene would drastically lower the rate of oligomerization. Therefore, the lower acetylene binding energy observed for the NiZn intermetallic compound explains the decrease in oligomerization and the resultant increase in both selectivity and stability.^157^

An extreme case of site isolation was reported by Furukawa *et al.*^159^ by the introduction of multiple metals to CsCl-type NiGa. The Ni and Ga sites were partially substituted by Fe/Cu and Ge, respectively, to form a high entropy intermetallic (HEI) of (NiFeCu)(GaGe). This HEI material was used as a catalyst for the hydrogenation of acetylene and demonstrated high selectivity for ethylene compared to the parent NiGa catalyst. These site-specific substitutions lowered the energy barrier of acetylene hydrogenation and weakened acetylene adsorption, facilitating hydrogen adsorption and activation. DFT studies demonstrated the most stable facet of the HEI was (110), because of the relaxation of the surface upon site-specific substitution. The lower surface energy of (110) promotes the desorption of ethylene, inhibiting undesired over-hydrogenation. DOS calculations indicated there was a negligible change in the electronic structure of Ni upon substitution with Fe and Cu, suggesting the enhanced catalytic performance was due to geometric effects (site isolation) rather than electronic effects.^159^

**Fig. 12:**
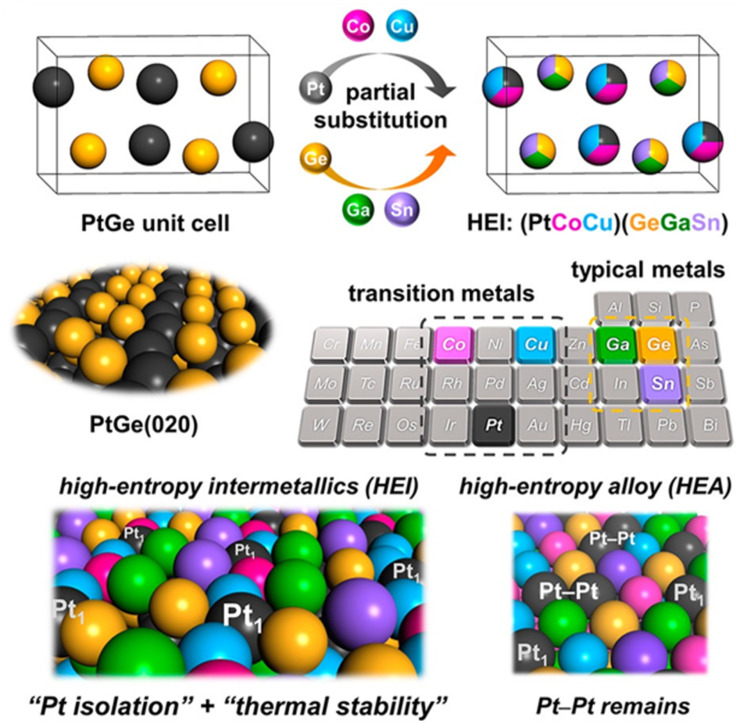
Schematic of the site-specific substitution of PtGe to form HEIs, and the Pt site isolation in HEIs compared with HEAs. Reprinted with permission from ref. 160, copyright 2022, the American Chemical Society.

Furukawa *et al.* also demonstrated the extreme site isolation achieved in HEIs through the site-specific substitution of the FeAs-type PtGe intermetallics.^160^ The Pt site in the binary PtGe was partially substituted by catalytically less active metals (Co and Cu), whereas the Ge site was partially replaced by inert metals (Ga and Sn) to form a senary HEI of the form, (PtCoCu)(GeGaSn) (Fig. 12). This HEI catalyst was used for propane dehydrogenation, demonstrating high selectivity to propylene by inhibiting unwanted side reactions. X-ray absorption near-edge structure (XANES) confirmed the d-band of Pt did not significantly change between PtGe and the HEI, indicating negligible electronic effects upon Pt substitution, consistent with DFT calculations (a very similar observation was reported by the authors in the case of (NiFeCu)(GaGe)).^159^ The high selectivity of the HEI was attributed to geometric effects due to the dilution of Pt with less active metals, which reduced the concentration of surface Pt–Pt ensembles and hence favored the desorption of propylene as a consequence of the limited number of sites available for binding propylene. The authors compared the HEI to other quaternary catalysts to demonstrate the significance of the high entropy effect on the catalyst selectivity and stability. However, it is very challenging to attribute such improvement to the high entropy effect and/or site isolation since the composition of the active metal (Ni/Pt) was not constant across these catalysts.

### 3.3 Role of electronic modification on the performance of intermetallic catalysts in selective hydrogenation reactions

The previous section discussed the precise control of the active site configuration and composition that can be achieved using intermetallic compounds and how this control affects the catalyst performance in selective hydrogenation reactions. However, intermetallic compounds also offer the opportunity to modify the electronic properties of active sites. While there are several examples of intermetallic compounds in which the dominant effect is geometric, there are few examples in which the dominant effect is electronic. Instead, the improvement in the catalytic performance of these intermetallic compounds can be attributed to a combination of electronic and geometric modifications. The ordered structures inherent to intermetallic compounds allow the contributions of these two effects to be distinguished in many cases, as discussed in the following section.

**Fig. 13:**
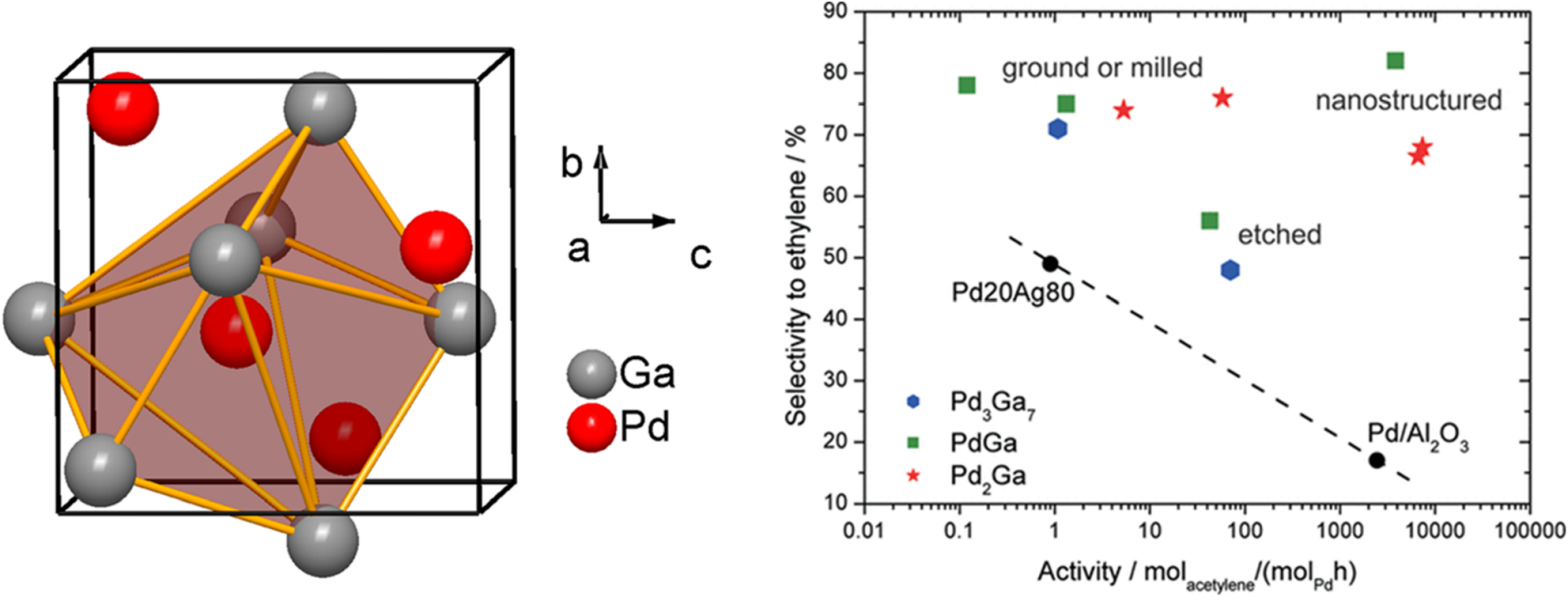
Isolation of Pd-sites in the structure of PdGa (left, made by DIAMOND 3, adapted from ref. 27), selectivity to ethylene in % over the rate of acetylene hydrogenation for PdGa, Pd_2_Ga, Pd_3_Ga_7_, and other industrially relevant catalysts (right, reprinted with permission from ref. 27, copyright 2010, the American Chemical Society).

Pd–Ga ordered intermetallic compounds (*e.g.* GaPd_2_, GaPd, Ga_7_Pd_3_) were proven to be highly selective for acetylene hydrogenation compared to pure Pd.^27,28,141,161^ The increased selectivity to ethylene over these intermetallic catalysts was largely explained based on the modification to the electronic structure of Pd through Pd–Ga interactions (Pd 4d–Ga 4p hybridization) (Section 2.6, Fig. 7); however, the isolation of Pd active sites by Ga may also play a role, and it is difficult to decouple these effects. Electronic structure calculations coupled with X-ray photoelectron spectroscopy (XPS) demonstrated a lower DOS at the Fermi level in PdGa compared to monometallic Pd, indicating an induced directionality in Pd–Ga interactions, *i.e.*, partial covalent character in the heteroatomic interactions. QTAIM analysis suggested the electronegativity difference between Pd and Ga is largely responsible for the electronic modification observed (Fig. 8). The partially negative Pd sites formed in intermetallic Pd–Ga enhance the tendency of acetylene to form a donor complex without being activated by backdonation through the Pd 4d-states (confirmed through infrared spectroscopic studies of CO adsorption), which leads to the superior selectivity to ethylene.^27,141^ The directionality in the Pd–Ga bonds is also an important factor in the increased stability observed for these catalysts and accounts for the increased resistance to segregation that frequently occurs in solid solution alloys. Despite the strong evidence that the electronic modifications significantly improved the selectivity and stability in Pd–Ga intermetallics, the Pd sites were completely isolated (Fig. 13). DFT studies revealed the configuration of the Pd active sites (Pd trimers or Pd monomers) affected the selectivity to ethylene.^162^ Both configurations were found to have similar barriers for acetylene hydrogenation; however, the Pd trimer sites were found to have a significantly higher barrier for ethylene hydrogenation, largely due to the hydrogen diffusion barrier to the active site, which would result in improved selectivity for Pd trimer sites.^162^ Therefore, the geometry of the active sites present in the different Pd–Ga intermetallic compounds also contributes to the selectivity of the catalyst during acetylene hydrogenation, meaning any improvements are likely due to a combination of electronic and geometric effects.

**Fig. 14:**
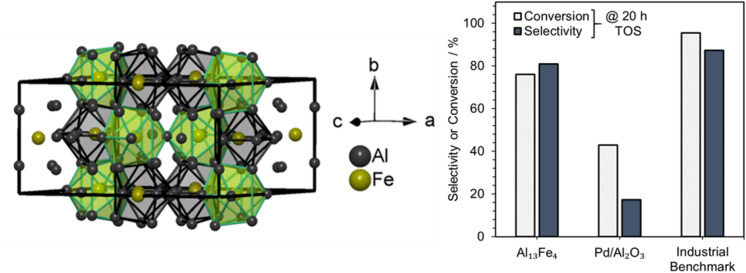
Isolation of Fe sites by Al in the bulk structure of Al_13_Fe_4_. Conversion (C) and selectivity (S) to ethylene of unsupported Al_13_Fe_4_, 5 wt% Pd/Al_2_O_3_ and an industrial benchmark catalyst for the selective hydrogenation of acetylene over 20 h time on stream. Adapted from ref. 163.

In the search for alternatives to expensive noble metals (*e.g.*, Pd), the unique bonding and control over the active site configuration suggest intermetallic compounds offer the opportunity to develop such catalysts. Armbrüster *et al.*^163^ introduced a novel PGM-free catalyst, Al_13_Fe_4_, for the selective hydrogenation of acetylene. Al_13_Fe_4_ crystallizes with the monoclinic space group *C*2/*m* with more than 100 atoms per unit cell. All Fe sites were coordinated by Al or consisted of Fe–Al–Fe ensembles embedded in the three-dimensional Al-network (Fig. 14). The covalent Fe–Al and Fe–Al–Fe multi-centered interactions lead to the site-isolation of Fe, which alters the electronic structure of the compound with respect to pure Fe and provides thermodynamic stability. The increased thermodynamic stability prevents segregation under the reaction conditions.^163^

An in-depth DFT (including surface energy calculations) and STM study of Al_13_Fe_4_ revealed the active sites of Al_13_Fe_4_ likely comprised isolated Fe atoms protruding from a pentagonal ensemble of Al atoms (*i.e.* Al_5_Fe), which were found to appear on the Al_13_Fe_4_ (010) surface.^164^ In other intermetallic catalysts, site isolation is crucial to achieving high selectivity in the hydrogenation of acetylene to ethylene. On completely isolated sites, acetylene adsorbs to the transition metal atoms within these ensembles *via* a di-σ configuration, but ethylene can only form a weak π-bond.^27,28,141,157,161^ The weaker binding of ethylene is crucial to preventing over-hydrogenation to ethane, while active site isolation minimizes oligomerization since the greater distances between active sites prevents coupling of hydrocarbon adsorbates. By taking advantage of the unique control over the active site composition through the use of intermetallic compounds, Armbrüster *et al.* demonstrated the development of noble metal free catalysts for selective hydrogenation reactions.^163^

Heusler alloys have recently attracted the attention of the catalysis community. Kojima *et al.*^86^ found that Co_2_MnGe and Co_2_FeGe alloys exhibit high alkene selectivity during alkyne hydrogenation. The catalytic performance of the alloys was tuned through the substitution of different elements in the parent compounds. The substitution of Ga into the Ge position of Co_2_FeGe led to the formation of isostructural Co_2_FeGa_0.5_Ge_0.5_, which showed high rates of acetylene hydrogenation with low ethylene selectivity. The authors proposed that based on apparent activation energies and DFT-based binding energy calculations, Ga has a higher propensity to adsorb small molecules (*e.g.*, C_2_H_4_, C_2_H_2_) than Ge, resulting in increased activity but lower selectivity. The substitution of Mn for Fe (Co_2_Mn_*x*_Fe_1−*x*_Ge) resulted in very high alkene selectivity even at complete conversion. The authors hypothesized this was largely due to a change in the electronic structure due to site-specific substitution. The results of this study highlight the flexibility and tunability of Heusler compounds, allowing for the alteration of the geometric ensembles and/or electronic structure to optimize catalytic performance.

Another strategy for the modification of the electronic structure of active sites was demonstrated by Niu *et al.*^165^ and Chen *et al.*^96^ through the introduction of carbon into the lattices of cubic Ni_3_Zn and Pd_3_Zn to form intermetallic carbides. The overall structure of the intermetallic compounds remained the same, and carbon atoms occupied interstitial sites. Through combined DFT and XANES experiments, the authors showed the C atom coordinates with the active metal site and neutralizes its positive charge. This process facilitates ethylene desorption and enhances acetylene hydrogenation selectivity.

Liu *et al.*^166^ identified a clear difference in acetylene hydrogenation activity between atomically ordered CsCl-type PdCu intermetallic nanoparticles and an fcc bimetallic solid solution of PdCu. The specific activity of CsCl-type PdCu was one order of magnitude greater than that of fcc-PdCu. CsCl-type PdCu is characterized by more densely populated and weaker Pd–Cu bonds than fcc-PdCu, along with a lower coordination number and higher-lying d-states. The authors argued the high-lying d-band centers facilitated hydrogen dissociation on the isolated Pd surface, leading to much higher activity for CsCl-type PdCu than for the substitutional solid solution (fcc-PdCu).^166^

### 3.4 Increasing the surface area of intermetallic compounds *via* alternative synthetic techniques

While the previous section mostly deals with the utilization of bulk intermetallic catalysts for the selective hydrogenation of acetylene, one of the challenges associated with bulk intermetallic compounds is their low surface area (typically <0.5 m^2^ g^−1^), which results in several fundamental and practical challenges, including limitations on able characterization techniques. While the preparation of nanoparticulate intermetallic catalysts can have significant downsides (*e.g.* homogeneity, support effects), they open the possibility to utilize additional characterization methods that can provide further experimental information regarding the active sites present in IMCs. The following section discusses intermetallic nanoparticle systems used for selective alkyne hydrogenation, focusing on the benefits and challenges associated with nanoparticulate IMCs.

Zhang *et al.*^167^ prepared ZnO-supported intermetallic PdZn nanoparticles (*P*4/*mmm*, CuTi-type), in which the active site was shown to consist of Pd–Zn–Pd ensembles. The authors studied the active site configuration using infrared spectroscopy of CO adsorption and measured the adsorption energies of relevant molecules on the active sites using microcalorimetry. Compared to the CO-FTIR spectrum obtained for a monometallic Pd catalyst, the spectrum for the PdZn catalyst showed a significant decrease in bridging CO, with the vast majority present as the linear bound form. This is consistent with the isolation of Pd sites since the increased distance between Pd sites in the Pd–Zn–Pd ensembles disfavors the formation of bridging CO. Additionally, a red shift in the wavenumber for the linear bound CO indicates increased electron density on Pd due to electron transfer from Zn to Pd. XPS analysis of the Pd–Zn catalyst was consistent with the IR results, finding a lower binding energy for Pd 3d_5/2_ in Pd–Zn compared to monometallic Pd. The higher surface area of the nanoparticulate catalyst also allowed the adsorption energy of relevant species, such as acetylene and ethylene, to be measured. The authors found the binding energies of acetylene on monometallic Pd and the Pd–Zn catalysts were comparable; however, the binding energy of ethylene was significantly lower on Pd–Zn/ZnO. The suppressed adsorption of ethylene on the Pd–Zn intermetallic catalyst was consistent with the experimentally observed improvement in ethylene selectivity during acetylene hydrogenation. DFT results suggest the site isolation of Pd in the Pd–Zn–Pd ensembles resulted in weakly π-bound ethylene, leading to improved ethylene selectivity. Electronic modifications may also contribute because the increased electron density on Pd is likely to result in weakened binding of electron-rich unsaturated molecules. Therefore, the improved selectivity observed during acetylene hydrogenation with the Pd–Zn intermetallic catalyst is likely due to a combination of geometric and electronic effects. Preparation of nanoparticulate intermetallic compounds has allowed for additional characterization techniques that are inaccessible with the low surface area of bulk intermetallics; however, it is important to be aware that confirming phase purity becomes more challenging since the smaller grain sizes broadened Bragg reflections in powder XRD.^167^

Another approach to increase the surface area of intermetallic compounds is through colloidal synthesis. Liu *et al.*^168^ synthesized Ni_*x*_M_*y*_ (M = Ga, Sn) intermetallic nanocrystals with various compositions and ordered crystal structures. Most of these compounds showed high alkene selectivity for alkyne hydrogenation, including acetylene. The authors reported the improvement was due to a combination of electronic modification and site isolation (*i.e.*, geometric effect); however, the origin of these effects and the direct implications of these modifications on catalysis were not explored in detail. Additionally, the presence of a residual capping agent (oleylamine, a long chain amine) has the potential to contribute to changes in the catalytic properties, including selectivity. Removal of oleylamine (and similar capping agents) from the surface of colloidal nanoparticles is known to be challenging, with the most successful methods often having inadvertent and potentially problematic consequences, *e.g.*, calcination,^169^ which is likely to result in oxidation of intermetallic compounds. Any changes in the measured catalytic behavior of intermetallic nanoparticles produced *via* colloidal methods may be due to the inherent properties of IMC or the presence of capping agents on the surface of the particles.

Furukawa *et al.*^170^ recently prepared supported nanoparticles of Cu-substituted L1_2_-type pseudo-binary compounds (Ni_1−*x*_Cu_*x*_)_3_Ga/TiO_2_ (*x* = 0.2, 0.25, 0.33, 0.5, 0.6, and 0.75) by conventional wet impregnation. The catalysts were characterized with STEM-EDX (scanning transmission electron microscopy coupled with energy-dispersive X-ray spectroscopy), pXRD and EXAFS to confirm the formation of the desired crystallographic phase. However, as discussed above, small impurities may be missed due to the broadened and weak signals in the pXRD patterns inherent to the small particles and/or low metal content of the supported catalysts. The catalysts were evaluated for the selective hydrogenation of acetylene and (Ni_0.8_Cu_0.2_)_3_Ga/TiO_2_ was found to be the optimum catalyst in terms of activity and selectivity. This highlights a key benefit of preparing intermetallic nanoparticles: high selectivity due to the controlled electronic and geometric modifications associated with IMC and high activity due to the increased metal surface area. DFT calculations indicated the increased selectivity was due to the formation of Ni_2_Cu hollow sites on the lowest energy (111) surface that destabilized the multifold coordination of adsorbates, leading to significantly higher adsorption energy of ethylidene compared to the Ni_3_ sites present on Ni_3_Ga and therefore prevented over-hydrogenation to ethane. Overall, intermetallic nanoparticles were found to be highly active and selective in acetylene hydrogenation; however, concerns over homogeneity implies the implementation of these catalysts as models to understand the fundamental origins of structure–activity–selectivity relationships is challenging.^170^

The difficulties in synthesizing phase-pure intermetallic nanoparticles are highlighted by Dasgupta *et al.*, who investigated the preparation of supported Pd–Zn, Cu–Zn and Ni–Zn intermetallic nanoparticles.^171^ The catalysts were synthesized by first preparing a M/SiO_2_ (where M = Pd, Ni or Cu) catalyst *via* standard impregnation techniques, followed by the vapor deposition of Zn, with the M : Zn molar ratio set to match the γ-brass phase. Powder XRD patterns of the supported nanoparticulate M–Zn catalysts confirmed the formation of the γ-brass phase, with no residual reflections corresponding to the parent metal observable. However, the atomic ratio of Pd : Zn in individual nanoparticles obtained through STEM-EDX analysis varied, with occasional particles having Pd : Zn atomic ratios outside the compositional bounds of the γ-brass phase. While these outliers may be in the minority, if the impurity phase is significantly more active than the target compound (*i.e.*, γ-brass phase), even a minority concentration would be expected to dominate the catalytic behavior. Additionally, as discussed above, small changes in the composition of Pd–Zn γ-brass compounds (*i.e.*, even changes to the Pd : Zn atomic ratio within the compositional bounds) have been shown to have dramatic effects on the activity and selectivity in acetylene hydrogenation.^140^ The authors used ethylene hydrogenation as a probe reaction for active site modification due to the formation of Pd–Zn γ-brass and found the apparent activation energy increased to 63 kJ mol^−1^, as compared to 38 kJ mol^−1^ for monometallic Pd.^171^ While the formation of Pd–Zn intermetallic compounds impacted the catalytic behavior, the variation in the composition of the Pd–Zn nanoparticles demonstrates it is not possible to relate this change to specific active site modifications, like in the case of bulk Pd–Zn γ-brass. The success of this method was largely dependent on the identity of M, with only M = Pd producing small nanoparticles (<10 nm) of the desired intermetallic compound. Additionally, the low boiling point of metallic Zn is crucial to this approach.^171^

Other approaches rely on the use of expensive and/or highly air sensitive reagents, *e.g.*, the co-reduction of ionic metal precursors to form GaPd and GaPd_2_ (ref. 172) or the thermal reduction of a zerovalent organozinc precursor in hot organoamine solvent to produce various M–Zn based intermetallics (PdZn, AuZn and Au_3_Zn, as well as several γ-brasses).^173^ Chemical vapor deposition of the organotin compound Sn(CH_3_)_4_ onto Ni/SiO_2_ was performed by Onda *et al.*^174^ to synthesize catalytically relevant Ni–Sn intermetallic nanocrystals. Furukawa *et al.*^175^ applied a rather simple co-impregnation method to synthesize SiO_2_-supported Heusler compound Co_2_FeGe, which was explored as a selective hydrogenation catalyst.

## 4. Conclusive remarks: a perspective on the future of intermetallics in heterogeneous catalysis

The most significant benefit offered by intermetallic compounds is the precise control over the active site, both in terms of structure and composition (geometric effects) and electronic structure modifications. Isolation of active sites by a more catalytically ‘inert’ element can result in active sites with ensembles of a specific geometry, while minute adjustments in the bulk stoichiometry of the material can vary the composition of exposed active sites. Differences in the coordination environment of catalytically active elements can result in changes to the bulk and surface electronic structure, with some systems displaying partial charge transfer or covalent and partially ionic type interactions in an overall metallic system. This control over active sites is what makes intermetallic compounds ideal as model catalysts; it enables in-depth structure–activity relationships to be established and facilitates detailed kinetics studies, such that it is possible to produce fully coverage-enumerated microkinetic models.^139^ Intermetallic compounds offer the unique opportunity to rationally design active, selective and stable catalysts.

However, there are limitations on the scope of reactions where intermetallic compounds are likely to remain active and stable, largely due to their susceptibility to oxidation. While this review has focused on the use of intermetallic compounds as catalysts for selective alkyne hydrogenations, there are many examples of their use in other reaction types;^176–179^ however, all of these reactions are carried out in reducing environments. When intermetallic compounds are used in processes with potentially oxidizing environments, such as the steam reforming of methanol, the catalyst is likely to be at least partially oxidized. Armbrüster *et al.*^180^ found the most active and selective PdZn-based catalyst consists of a combination of reduced and oxidized species. Therefore, although there are several processes in which intermetallic compounds can be employed as model catalysts, even mildly oxidizing environments can modify the catalyst and either introduce additional complexity or render it inactive.

One consequence of the unique bonding and interactions present in intermetallic compounds is their potential to act as ‘pseudoelements’,^11^ where the electronic structure of the intermetallic compound mimics that of another element. This opens opportunities to develop catalysts made of cheaper and more abundant elements to replace current catalysts that often consist of rarer and more expensive elements such as Pd, Pt and other noble metals. A proof-of-concept example explored PdZn and PdCd intermetallic compounds for the steam reforming of methanol and found that both the electronic structure and catalytic performance of the materials were similar to those of a monometallic copper catalyst.^11^ While this is a potentially exciting avenue to explore in the effort to develop active and stable noble-metal-free catalysts, it is important to consider the challenges associated with the use of intermetallic compounds for large-scale industrial processes; their low surface area (and therefore poor metal utilization) and the feasibility of scaling up solid state synthetic methods (synthesis under vacuum at high temperatures for extended periods). Therefore, if the full potential of intermetallic compounds is to be realized, substantial effort would be required to develop simple and reliable methods for the synthesis of supported versions of these compounds, which would make them more viable as catalysts for industrial processes.

Ultimately, bulk intermetallic compounds allow significant control and flexibility over the active sites, which makes it possible to rationally design specific active sites for a chosen reaction, making them ideal as model catalysts. If the challenges associated with low surface area and challenging synthetic methods can be overcome, this would expand the scope of applications for intermetallic compounds and allow their unique properties to be fully leveraged.

## Data availability

This review utilized data available and cited in the literature. New data were generated solely for illustrative purposes (including all structural figures, particularly Fig. 7 and 8). Data will be made available upon request.

## Author contributions

N. R. and G. C. conceptualized the initial draft of the manuscript. N. R. and K. M. co-wrote the original draft, prepared the structural figures for visualization, and conducted the formal analysis. M. E. contributed to writing, reviewing, editing, and visualization. G. C. revised and edited the manuscript. R. M. R. revised and edited the manuscript and supervised the entire project.

## Conflicts of interest

There are no conflicts of interest to declare.

## References

[cit1] Armbrüster M. (2020). Intermetallic compounds in catalysis – a versatile class of materials meets interesting challenges. Sci. Technol. Adv. Mater..

[cit2] Armbrüster M., Schlögl R., Grin Y. (2014). Intermetallic compounds in heterogeneous catalysis—a quickly developing field. Sci. Technol. Adv. Mater..

[cit3] Bauer J. C., Chen X., Liu Q., Phan T.-H., Schaak R. E. (2008). Converting nanocrystalline metals into alloys and intermetallic compounds for applications in catalysis. J. Mater. Chem..

[cit4] Dasgupta A., Rioux R. M. (2019). Intermetallics in catalysis: An exciting subset of multimetallic catalysts. Catal. Today.

[cit5] Furukawa S., Komatsu T. (2017). Intermetallic Compounds: Promising Inorganic Materials for Well-Structured and Electronically Modified Reaction Environments for Efficient Catalysis. ACS Catal..

[cit6] Li J., Sun S. (2019). Intermetallic Nanoparticles: Synthetic Control and Their Enhanced Electrocatalysis. Acc. Chem. Res..

[cit7] Nakaya Y., Furukawa S. (2024). High-entropy intermetallics: emerging inorganic materials for designing high-performance catalysts. Chem. Sci..

[cit8] Penner S., Kheyrollahi Nezhad P. D. (2021). Steering the Catalytic Properties of Intermetallic Compounds and Alloys in Reforming Reactions by Controlled in Situ Decomposition and Self-Activation. ACS Catal..

[cit9] Quilis C., Mota N., Millán E., Pawelec B., Navarro Yerga R. M. (2024). Application of Intermetallic Compounds as Catalysts for the Selective Hydrogenation of CO2 to Methanol. ChemCatChem.

[cit10] Rößner L., Armbrüster M. (2019). Electrochemical Energy Conversion on Intermetallic Compounds: A Review. ACS Catal..

[cit11] Tsai A. P., Kameoka S., Nozawa K., Shimoda M., Ishii Y. (2017). Intermetallic: A Pseudoelement for Catalysis. Acc. Chem. Res..

[cit12] Bos A. N. R., Westerterp K. R. (1993). Mechanism and kinetics of the selective hydrogenation of ethyne and ethene. Chem. Eng. Process. Process Intensif..

[cit13] Takht Ravanchi M., Sahebdelfar S., Komeili S. (2018). Acetylene selective hydrogenation: a technical review on catalytic aspects. Rev. Chem. Eng..

[cit14] Gardner L. (2005). The use of stainless steel in structures. Prog. Struct. Eng. Mater..

[cit15] Naboychenko S. S. , Murashova I. B. and Neikov O. D., in Handbook of Non-Ferrous Metal Powders, ed. O. D. Neikov, S. S. Naboychenko, I. V. Murashova, V. G. Gopienko, I. V. Frishberg and D. V. Lotsko, Elsevier, Oxford, 2009, pp. 331–368, 10.1016/B978-1-85617-422-0.00016-1

[cit16] Jozwik P., Polkowski W., Bojar Z. (2015). Applications of Ni3Al based intermetallic alloys—current stage and potential perceptivities. Materials.

[cit17] Paul A. R., Mukherjee M., Singh D. (2022). A Critical Review on the Properties of Intermetallic Compounds and Their Application in the Modern Manufacturing. Cryst. Res. Technol..

[cit18] Darolia R. (1991). NiAl alloys for high-temperature structural applications. JOM.

[cit19] Gourdon O., Gout D., Williams D. J., Proffen T., Hobbs S., Miller G. J. (2007). Atomic Distributions in the γ-Brass Structure of the Cu−Zn System: A Structural and Theoretical Study. Inorg. Chem..

[cit20] Brandenburg K. and Putz H., Crystal Impact GbR, Bonn, Germany, 2005

[cit21] Villars P. and Cenzual K., Pearson's crystal data: crystal structure database for inorganic compounds, 2007

[cit22] Krauss G. , Steels: processing, structure, and performance, ASM international, 2015

[cit23] Hume-Rothery W. , Researches on the nature, properties, and conditions of formation of intermetallic compounds, with special reference to certain compounds of tin, 1926

[cit24] Hume-Rothery W. (1967). Phase stability in metals and alloys. Springer Ser. Biomater. Sci. Eng..

[cit25] Mizutani U. (2012). Hume-Rothery rules for structurally complex alloy phases. MRS Bull..

[cit26] Kanatzidis M. G., Pöttgen R., Jeitschko W. (2005). The Metal Flux: A Preparative Tool for the Exploration of Intermetallic Compounds. Angew. Chem., Int. Ed..

[cit27] Armbrüster M., Kovnir K., Behrens M., Teschner D., Grin Y., Schlögl R. (2010). Pd−Ga Intermetallic Compounds as Highly Selective Semihydrogenation Catalysts. J. Am. Chem. Soc..

[cit28] Osswald J., Giedigkeit R., Jentoft R. E., Armbrüster M., Girgsdies F., Kovnir K., Ressler T., Grin Y., Schlögl R. (2008). Palladium–gallium intermetallic compounds for the selective hydrogenation of acetylene: Part I: Preparation and structural investigation under reaction conditions. J. Catal..

[cit29] Werwein A., Maaß F., Dorsch L. Y., Janka O., Pöttgen R., Hansen T. C., Kimpton J., Kohlmann H. (2017). Hydrogenation Properties of Laves Phases LnMg2 (Ln = La, Ce, Pr, Nd, Sm, Eu, Gd, Tb, Ho, Er, Tm, Yb). Inorg. Chem..

[cit30] Zeier W. G., Schmitt J., Hautier G., Aydemir U., Gibbs Z. M., Felser C., Snyder G. J. (2016). Engineering half-Heusler thermoelectric materials using Zintl chemistry. Nat. Rev. Mater..

[cit31] Amon A., Ormeci A., Bobnar M., Akselrud L. G., Avdeev M., Gumeniuk R., Burkhardt U., Prots Y., Hennig C., Leithe-Jasper A., Grin Y. (2018). Cluster Formation in the Superconducting Complex Intermetallic Compound Be21Pt5. Acc. Chem. Res..

[cit32] Cava R. J., Takagi H., Zandbergen H. W., Krajewski J. J., Peck W. F., Siegrist T., Batlogg B., van Dover R. B., Felder R. J., Mizuhashi K., Lee J. O., Eisaki H., Uchida S. (1994). Superconductivity in the quaternary intermetallic compounds LnNi2B2C. Nature.

[cit33] Provino A., Smetana V., Paudyal D., Gschneidner K. A., Mudring A.-V., Pecharsky V. K., Manfrinetti P., Putti M. (2016). Gd3Ni2 and Gd3CoxNi2−x: magnetism and unexpected Co/Ni crystallographic ordering. J. Mater. Chem. C.

[cit34] Steurer W. and Dshemuchadse J., Intermetallics: structures, properties, and statistics, Oxford University Press, 2016

[cit35] Schäfer H. (1985). On the Problem of Polar Intermetallic Compounds: The Stimulation of E. Zintl's Work for the Modern Chemistry of Intermetallics. Annu. Rev. Mater. Sci..

[cit36] Jain A., Ong S. P., Hautier G., Chen W., Richards W. D., Dacek S., Cholia S., Gunter D., Skinner D., Ceder G., Persson K. A. (2013). Commentary: The Materials Project: A materials genome approach to accelerating materials innovation. APL Mater..

[cit37] Merchant A., Batzner S., Schoenholz S. S., Aykol M., Cheon G., Cubuk E. D. (2023). Scaling deep learning for materials discovery. Nature.

[cit38] Chanussot L., Das A., Goyal S., Lavril T., Shuaibi M., Riviere M., Tran K., Heras-Domingo J., Ho C., Hu W., Palizhati A., Sriram A., Wood B., Yoon J., Parikh D., Zitnick C. L., Ulissi Z. (2021). Open Catalyst 2020 (OC20) Dataset and Community Challenges. ACS Catal..

[cit39] Nesper R. (1991). Bonding patterns in intermetallic compounds. Angew Chem. Int. Ed. Engl..

[cit40] Iandelli A. and Palenzona A., Crystal chemistry of intermetallic compounds, Handbook on the physics and chemistry of rare earths, 1979, vol. 2, pp. 1–54

[cit41] Ferro R., Saccone A. (1996). Structure of intermetallic compounds and phases. Phys. Metall..

[cit42] Kraus J. D., Van Buskirk J. S., Fredrickson D. C. (2023). The Zintl Concept Applied to Intergrowth Structures: Electron-Hole Matching, Stacking Preferences, and Chemical Pressures in Pd5InAs. Z. Anorg. Allg. Chem..

[cit43] Lim A., Fredrickson D. C. (2023). Entropic Control of Bonding, Guided by Chemical Pressure: Phase Transitions and 18-n+m Isomerism of IrIn3. Inorg. Chem..

[cit44] Zhang W., Li F., Li Y., Song A., Yang K., Wu D., Shang W., Yao Z., Gao W., Deng T., Wu J. (2024). The role of surface substitution in the atomic disorder-to-order phase transition in multi-component core–shell structures. Nat. Commun..

[cit45] Brese N. E., von Schnering H. G. (1994). Bonding trends in pyrites and a reinvestigation of the structures of PdAs2, PdSb2, PtSb2 and PtBi2. Z. Anorg. Allg. Chem..

[cit46] Gourdon O., Miller G. J. (2006). Intergrowth Compounds in the Zn-Rich Zn−Pd System: Toward 1D Quasicrystal Approximants. Chem. Mater..

[cit47] Lidin S., Folkers L. C. (2018). In Situ Synthesis and Single Crystal Synchrotron X-ray Diffraction Study of ht-Sn3Sb2: An Example of How Complex Modulated Structures Are Becoming Generally Accessible. Acc. Chem. Res..

[cit48] Kamp K. R., Fredrickson D. C. (2021). Frustrated Packing in Simple Structures: Chemical Pressure Hindrance to Isolobal Bonds in the TiAl3 type and ZrAl2.6Sn0.4. Inorg. Chem..

[cit49] Kuila S. K., Harshit, Roy N., Ghanta S., Pan R., Buxi K., Pramanik P., Bera A. K., Saha B., Yusuf S. M., Petříček V., Roy A., Jana P. P. (2023). Ni3InSb: Synthesis, Crystal Structure, Electronic Structure, and Magnetic Properties. Inorg. Chem..

[cit50] Roy N., Kuila S. K., Harshit, Pramanik P., Jana P. P. (2022). Selective Chemical Substitution of Cu in the Structure of TiAl3 Type InPd3: Experimental and Theoretical Studies. Eur. J. Inorg. Chem..

[cit51] Verniere A., Diop L. V. B., Sarr I., Schweitzer T., Malaman B. (2024). Crystal structure of new quaternary intermetallic compounds R2MoSi2C (R = Y, Gd). Acta Crystallogr., Sect. B.

[cit52] Latturner S. E., Bilc D., Mahanti S. D., Kanatzidis M. G. (2002). Quaternary Intermetallics Grown from Molten Aluminum: The Homologous Series Th2(AuxSi1-x)[AuAl2]nSi2 (n = 1, 2, 4). Chem. Mater..

[cit53] Reitz L. S., Hempelmann J., Müller P. C., Dronskowski R., Steinberg S. (2024). Bonding Analyses in the Broad Realm of Intermetallics: Understanding the Role of Chemical Bonding in the Design of Novel Materials. Chem. Mater..

[cit54] Friedrich M., Ormeci A., Grin Y., Armbrüster M. (2010). PdZn or ZnPd: Charge Transfer and Pd–Pd Bonding as the Driving Force for the Tetragonal Distortion of the Cubic Crystal Structure. Z. Anorg. Allg. Chem..

[cit55] Oliynyk A. O., Mar A. (2018). Discovery of Intermetallic Compounds from Traditional to
Machine-Learning Approaches. Acc. Chem. Res..

[cit56] Roy N., Giri S. (2024). Influence of the Homoatomic and Multicenter Bonding on the Formation of CsCl-type RhCd. Z. Anorg. Allg. Chem..

[cit57] Gross N., Kotzyba G., Künnen B., Jeitschko W. (2001). Binary Compounds of Rhodium and Zinc: RhZn, Rh2Zn11, and RhZn13. Z. Anorg. Allg. Chem..

[cit58] Oliynyk A. O., Antono E., Sparks T. D., Ghadbeigi L., Gaultois M. W., Meredig B., Mar A. (2016). High-Throughput Machine-Learning-Driven Synthesis of Full-Heusler Compounds. Chem. Mater..

[cit59] Gvozdetskyi V., Selvaratnam B., Oliynyk A. O., Mar A. (2023). Revealing Hidden Patterns through Chemical Intuition and Interpretable Machine Learning: A Case Study of Binary Rare-Earth Intermetallics RX. Chem. Mater..

[cit60] Sikdar R., Selvaratnam B., Mishra V., Mar A. (2025). Is There a Simple Descriptor to Predict Laves Phases?. Cryst. Growth Des..

[cit61] Sikdar R., Roy N., Selvaratnam B., Mishra V., Mumbaraddi D., Mondal A., Buxi K., Jana P. P., Mar A. (2024). Ahead by a Century: Discovery of Laves Phases Assisted by Machine Learning. Inorg. Chem..

[cit62] Selvaratnam B., Oliynyk A. O., Mar A. (2023). Interpretable Machine Learning in Solid-State Chemistry, with Applications to Perovskites, Spinels, and Rare-Earth Intermetallics: Finding Descriptors Using Decision Trees. Inorg. Chem..

[cit63] Oviedo F., Ferres J. L., Buonassisi T., Butler K. T. (2022). Interpretable and Explainable Machine Learning for Materials Science and Chemistry. Acc. Mater. Res..

[cit64] Fredrickson D. C., Miller G. J. (2018). Intermetallic Chemistry: New Advances in Humanity’s Age-Old Exploration of Metals and Alloys. Acc. Chem. Res..

[cit65] Hoffmann R. (1987). How Chemistry and Physics Meet in the Solid State. Angew Chem. Int. Ed. Engl..

[cit66] Miller G. J. (1998). The “Coloring Problem” in Solids: How It Affects Structure, Composition and Properties. Eur. J. Inorg. Chem..

[cit67] Mizutani U., Sato H., Massalski T. B. (2021). The original concepts of the Hume-Rothery rule extended to alloys and compounds whose bonding is metallic, ionic, or covalent, or a changing mixture of these. Prog. Mater. Sci..

[cit68] Mizutani U., Sato H. (2017). The Physics of the Hume-Rothery Electron Concentration Rule. Crystals.

[cit69] Hoistad L. M., Lee S. (1991). The Hume-Rothery electron concentration rules and second moment scaling. J. Am. Chem. Soc..

[cit70] Zintl E., Dallenkopf W. (1932). Über den Gitterbau von NaTl und seine Beziehung zu den Strukturen vom Typus des β-Messings, 4. Mitteilung über Metalle und Legierungen. Z. Phys. Chem..

[cit71] Wang F., Miller G. J. (2011). Revisiting the Zintl–Klemm Concept: Alkali Metal Trielides. Inorg. Chem..

[cit72] Tiefenthaler S., Korber N., Gärtner S. (2019). Synthesis of the Tetragonal Phase of Zintl’s NaTl and Its Structure Determination from Powder Diffraction Data. Materials.

[cit73] Smetana V., Rhodehouse M., Meyer G., Mudring A.-V. (2017). Gold Polar Intermetallics: Structural Versatility through Exclusive Bonding Motifs. Acc. Chem. Res..

[cit74] Lin Q., Miller G. J. (2018). Electron-Poor Polar Intermetallics: Complex Structures, Novel Clusters, and Intriguing Bonding with Pronounced Electron Delocalization. Acc. Chem. Res..

[cit75] Johnston R. L., Hoffmann R. (1992). Structure-Bonding Relationships in the Laves Phases. Z. Anorg. Allg. Chem..

[cit76] Laves F. and Löhberg K., Die Kristallstruktur von intermetallischen Verbindungen der Formel AB− 2, von, Weidmann, 1934

[cit77] Friauf J. B. (1927). The crystal structures of two intermetallic compounds. J. Am. Chem. Soc..

[cit78] Stein F., Palm M., Sauthoff G. (2005). Structure and stability of Laves phases part II—structure type variations in binary and ternary systems. Intermetallics.

[cit79] Stein F., Palm M., Sauthoff G. (2004). Structure and stability of Laves phases. Part I. Critical assessment of factors controlling Laves phase stability. Intermetallics.

[cit80] Ormeci A., Simon A., Grin Y. (2010). Structural topology and chemical bonding in Laves phases. Angew. Chem..

[cit81] Yartys V. A., Lototskyy M. V. (2022). Laves type intermetallic compounds as hydrogen storage materials: A review. J. Alloys Compd..

[cit82] Heusler F. (1903). Über magnetische manganlegierungen. Verh. Dtsch. Phys. Ges..

[cit83] Kawasaki J. K., Chatterjee S., Canfield P. C., Guest E. (2022). Full and half-Heusler compounds. MRS Bull..

[cit84] Manna K., Sun Y., Muechler L., Kübler J., Felser C. (2018). Heusler, Weyl and Berry. Nat. Rev. Mater..

[cit85] Elphick K., Frost W., Samiepour M., Kubota T., Takanashi K., Sukegawa H., Mitani S., Hirohata A. (2021). Heusler alloys for spintronic devices: review on recent development and future perspectives. Sci. Technol. Adv. Mater..

[cit86] Kojima T., Kameoka S., Fujii S., Ueda S., Tsai A.-P. (2018). Catalysis-tunable Heusler alloys in selective hydrogenation of alkynes: A new potential for old materials. Sci. Adv..

[cit87] Kojima T., Kameoka S., Tsai A.-P. (2019). The emergence of Heusler alloy catalysts. Sci. Technol. Adv. Mater..

[cit88] Wu Z., Ma S., Weng S., Liu H., Jin Z., Jiang X. (2024). Mechanistic Insights into the Direct Deoxygenation of Phenolic Compounds over Novel Heusler Alloy Catalysts. ACS Appl. Mater. Interfaces.

[cit89] Jin T., Jung Y. (2022). Classifying Intermetallic Tetragonal Phase of All-d-Metal Heusler Alloys for Catalysis Applications. Top. Catal..

[cit90] Mizutani U., Noritake T., Ohsuna T., Takeuchi T. (2010). Hume-Rothery electron concentration rule across a whole solid solution range in a series of gamma-brasses in Cu–Zn, Cu–Cd, Cu–Al, Cu–Ga, Ni–Zn and Co–Zn alloy systems. Philos. Mag..

[cit91] Roy N., Harshit, Mondal A., Wang F., Jana P. P. (2022). Structural and Theoretical Investigations on the Unique Coloring Scheme of the γ-Brass Type Phase: Cu5+δCd8-δ (−1.0≤δ≤0.1). Z. Anorg. Allg. Chem..

[cit92] Urban K., Feuerbacher M. (2004). Structurally complex alloy phases. J. Non-Cryst. Solids.

[cit93] Cahn J. W., Gratias D., Shechtman D. (1986). Indexing of icosahedral quasiperiodic crystals. J. Mater. Res..

[cit94] Samson S. (1962). Crystal Structure of NaCd2. Nature.

[cit95] Hafner J., Krajčí M. (2014). Surfaces of Complex Intermetallic Compounds: Insights from Density Functional Calculations. Acc. Chem. Res..

[cit96] Chen H., Li L., Zhao Z.-J., Yang B., Zhang Y., Liu X., Gu Q., Yu Z., Yang X., Gong J., Wang A., Zhang T. (2024). Co-infiltration and dynamic formation of Pd3ZnCx intermetallic carbide by syngas boosting selective hydrogenation of acetylene. Nat. Commun..

[cit97] Matar S. F. (2010). Intermetallic hydrides: A review with ab initio aspects. Prog. Solid State Chem..

[cit98] Tsai M.-H. (2016). Three Strategies for the Design of Advanced High-Entropy Alloys. Entropy.

[cit99] Yeh J. W., Chen S. K., Lin S. J., Gan J. Y., Chin T. S., Shun T. T., Tsau C. H., Chang S. Y. (2004). Nanostructured High-Entropy Alloys with Multiple Principal Elements: Novel Alloy Design Concepts and Outcomes. Adv. Eng. Mater..

[cit100] Cantor B., Chang I. T. H., Knight P., Vincent A. J. B. (2004). Microstructural development in equiatomic multicomponent alloys. Mater. Sci. Eng., A.

[cit101] George E. P., Raabe D., Ritchie R. O. (2019). High-entropy alloys. Nat. Rev. Mater..

[cit102] Nakaya Y., Furukawa S. (2023). Catalysis of Alloys: Classification, Principles, and Design for a Variety of Materials and Reactions. Chem. Rev..

[cit103] Burdett J. K., Lee S., McLarnan T. J. (1985). Coloring problem. J. Am. Chem. Soc..

[cit104] Hohenberg P., Kohn W. (1964). Density functional theory (DFT). Phys. Rev..

[cit105] Löwdin P. O. (1950). On the non-orthogonality problem connected with the use of atomic wave functions in the theory of molecules and crystals. J. Chem. Phys..

[cit106] Mulliken R. S. (1955). Electronic population analysis on LCAO–MO molecular wave functions. I. J. Chem. Phys..

[cit107] Gimarc B. M. (1983). Topological charge stabilization. J. Am. Chem. Soc..

[cit108] Roy N. (2023). A Theoretical Study on the Unique Site Preference and Atomic Ordering of Hexagonal CoSn-type Ternary Intermetallic Compound Co3Ge2Sn. Z. Anorg. Allg. Chem..

[cit109] Dronskowski R., Bloechl P. E. (1993). Crystal orbital Hamilton populations (COHP): energy-resolved visualization of chemical bonding in solids based on density-functional calculations. J. Phys. Chem..

[cit110] https://www.cohp.de/

[cit111] Maintz S., Deringer V. L., Tchougréeff A. L., Dronskowski R. (2013). Analytic projection from plane-wave and PAW wavefunctions and application to chemical-bonding analysis in solids. J. Comput. Chem..

[cit112] Deringer V. L., Tchougréeff A. L., Dronskowski R. (2011). Crystal Orbital Hamilton Population (COHP) Analysis As Projected from Plane-Wave Basis Sets. J. Phys. Chem. A.

[cit113] Müller P. C., Ertural C., Hempelmann J., Dronskowski R. (2021). Crystal Orbital Bond Index: Covalent Bond Orders in Solids. J. Phys. Chem. C.

[cit114] Krier G. , Jepsen O., Burkhardt A. and Andersen O. K., The TB-LMTO-ASA program, Stuttgart, 1995

[cit115] Nelson R., Ertural C., George J., Deringer V. L., Hautier G., Dronskowski R. (2020). LOBSTER: Local orbital projections, atomic charges, and chemical-bonding analysis from projector-augmented-wave-based density-functional theory. J. Comput. Chem..

[cit116] Giannozzi P., Baroni S., Bonini N., Calandra M., Car R., Cavazzoni C., Ceresoli D., Chiarotti G. L., Cococcioni M., Dabo I., Dal Corso A., de Gironcoli S., Fabris S., Fratesi G., Gebauer R., Gerstmann U., Gougoussis C., Kokalj A., Lazzeri M., Martin-Samos L., Marzari N., Mauri F., Mazzarello R., Paolini S., Pasquarello A., Paulatto L., Sbraccia C., Scandolo S., Sclauzero G., Seitsonen A. P., Smogunov A., Umari P., Wentzcovitch R. M. (2009). QUANTUM ESPRESSO: a modular and open-source software project for quantum simulations of materials. J. Phys.: Condens. Matter.

[cit117] Eck B. , wxDragon 2.2.2, RWTH Aachen University, Germany, 2020

[cit118] Armbrüster M., Borrmann H., Wedel M., Prots Y., Giedigkeit R., Gille P. (2010). Refinement of the crystal structure of palladium gallium (1:1), PdGa. Z. Kristallogr. New Cryst. Struct..

[cit119] Chen D., Yu R., Zhao H., Jiao J., Mu X., Yu J., Mu S. (2024). Boron-Induced Interstitial Effects Drive Water Oxidation on Ordered Ir−B Compounds. Angew. Chem., Int. Ed..

[cit120] Liang J., Pan X., Zeng B., Zhong C., Zhang L., Zhang J., Song H., Du L., Liao S., Cui Z. (2025). Achieving Highly Durable Intermetallic PtMn3N Electrocatalyst via the Strong Metal-N Bonds toward Oxygen Reduction Reaction. Adv. Funct. Mater..

[cit121] Wang H., Zou L., Li M., Zhang L. (2023). Identification of linear scaling relationships in polysulfide conversion on α-In2Se3-supported single-atom catalysts. Phys. Chem. Chem. Phys..

[cit122] Bader R. F. W. and Nguyen-Dang T. T., in Adv. Quantum Chem., Elsevier, 1981, vol. 14, pp. 63–124

[cit123] https://www.chemistry.mcmaster.ca/bader/

[cit124] Tang W., Sanville E., Henkelman G. (2009). A grid-based Bader analysis algorithm without lattice bias. J. Phys.: Condens. Matter.

[cit125] Savin A., Nesper R., Wengert S., Fässler T. F. (1997). ELF: The Electron Localization Function. Angew Chem. Int. Ed. Engl..

[cit126] Kohout M. (2004). A measure of electron localizability. Int. J. Quantum Chem..

[cit127] Agnarelli L., Prots Y., Ramlau R., Schmidt M., Burkhardt U., Leithe-Jasper A., Grin Y. (2022). Mg29–xPt4+y: Chemical Bonding Inhomogeneity and Structural Complexity. Inorg. Chem..

[cit128] Fredrickson D. C. (2012). DFT-Chemical Pressure Analysis: Visualizing the Role of Atomic Size in Shaping the Structures of Inorganic Materials. J. Am. Chem. Soc..

[cit129] https://www2.chem.wisc.edu/∼danny/software/dftcpp/

[cit130] Lim A., Fredrickson D. C. (2024). Navigating the 18-n+m Isomers of PdSn2: Chemical Pressure Relief through Isolobal Bonds and Main Group Clustering. Inorg. Chem..

[cit131] Sanders K. M., Gressel D. G., Fredrickson R. T., Fredrickson D. C. (2024). Toward Predicting the Assembly of Modular Intermetallics from Chemical Pressure Analysis: The Interface Nucleus Approach. Inorg. Chem..

[cit132] Roy N. (2024). A Fundamental Perspective on the Selective Zn Substitution in Ternary Ordered Intermetallic Compound Cu6Zn2Sb2. Z. Anorg. Allg. Chem..

[cit133] Misra S., Mallick S., Koley B., Wang F., Chatterjee S., Jana P. P. (2020). Chemical substitution of Zn in the structure of ordered Cu6Zn2Sb2: A structural and theoretical study. Solid State Sci..

[cit134] Kuila S. K., Pramanik P., Roy N., Buxi K., Bera A. K., Jana P. P. (2025). Unprecedented Site Preference of Sn in the Structure of CoSn-Type NiIn1–xSnx (x < 0.7). Inorg. Chem..

[cit135] Wuttig M., Schön C.-F., Kim D., Golub P., Gatti C., Raty J.-Y., Kooi B. J., Pendás Á. M., Arora R., Waghmare U. (2024). Metavalent or Hypervalent Bonding: Is There a Chance for Reconciliation?. Adv. Sci..

[cit136] Müller P. C., Elliott S. R., Dronskowski R., Jones R. O. (2024). Chemical bonding in phase-change chalcogenides. J. Phys.: Condens. Matter.

[cit137] Spanjers C. S., Dasgupta A., Kirkham M., Burger B. A., Kumar G., Janik M. J., Rioux R. M. (2017). Determination of bulk and surface atomic arrangement in Ni–Zn γ-brass phase at different Ni to Zn ratios. Chem. Mater..

[cit138] Gong R., Dasgupta A., Shang S.-L., He H., Kirkham M., Zimmerer E. K., Canning G. A., Janik M. J., Rioux R. M., Liu Z.-K. (2025). Determination of Site Occupancy in the M–Pd–Zn (M = Cu, Ag, and Au) γ-Brass Phase by CALculation of PHAse Diagrams Modeling and Rietveld Refinement. Inorg. Chem..

[cit139] He H., Canning G. A., Nguyen A., Dasgupta A., Meyer R. J., Rioux R. M., Janik M. J. (2023). Active-site isolation in intermetallics enables precise identification of elementary reaction kinetics during olefin hydrogenation. Nat. Catal..

[cit140] Dasgupta A., He H., Gong R., Shang S.-L., Zimmerer E. K., Meyer R. J., Liu Z.-K., Janik M. J., Rioux R. M. (2022). Atomic control of active-site ensembles in ordered alloys to enhance hydrogenation selectivity. Nat. Chem..

[cit141] Kovnir K., Armbrüster M., Teschner D., Venkov T. V., Szentmiklósi L., Jentoft F. C., Knop-Gericke A., Grin Y., Schlögl R. (2009). In situ surface characterization of the intermetallic compound PdGa – A highly selective hydrogenation catalyst. Surf. Sci..

[cit142] Molnár Á., Sárkány A., Varga M. (2001). Hydrogenation of carbon–carbon multiple bonds: chemo-, regio- and stereo-selectivity. J. Mol. Catal. A: Chem..

[cit143] Sabatier P. , La catalyse en chimie organique, ed. C. Béranger, 1920

[cit144] Frevel L. K. and Kressley L. J., US Pat., US2802889A, 1957

[cit145] Johnson M. M. , Walker D. W. and Nowack G. P., US Pat., US4404124A, 1983

[cit146] Johnson M. M. , Walker D. W. and Nowack G. P., US Pat., US4484015A, 1984

[cit147] Zea H., Lester K., Datye A. K., Rightor E., Gulotty R., Waterman W., Smith M. (2005). The influence of Pd–Ag catalyst restructuring on the activation energy for ethylene hydrogenation in ethylene–acetylene mixtures. Appl. Catal., A.

[cit148] Huang D. C., Chang K. H., Pong W. F., Tseng P. K., Hung K. J., Huang W. F. (1998). Effect of Ag-promotion on Pd catalysts by XANES. Catal. Lett..

[cit149] Meitzner G., Sinfelt J. H. (1994). X-ray absorption studies of the electronic structures of Pd-Ag and Pd-Au alloys. Catal. Lett..

[cit150] Cordts B., Pease D., Azároff L. V. (1981). Difference between the local density of unoccupied states in Ag-Pd and Cu-Ni alloys. Phys. Rev. B: Condens. Matter Mater. Phys..

[cit151] Jin Y., Datye A. K., Rightor E., Gulotty R., Waterman W., Smith M., Holbrook M., Maj J., Blackson J. (2001). The Influence of Catalyst Restructuring on the Selective Hydrogenation of Acetylene to Ethylene. J. Catal..

[cit152] O'Connor C. R., van Spronsen M. A., Egle T., Xu F., Kersell H. R., Oliver-Meseguer J., Karatok M., Salmeron M., Madix R. J., Friend C. M. (2020). Hydrogen migration at restructuring palladium–silver oxide boundaries dramatically enhances reduction rate of silver oxide. Nat. Commun..

[cit153] Vignola E., Steinmann S. N., Vandegehuchte B. D., Curulla D., Sautet P. (2016). C2H2-Induced Surface Restructuring of Pd–Ag Catalysts: Insights from Theoretical Modeling. J. Phys. Chem. C.

[cit154] Studt F., Abild-Pedersen F., Bligaard T., Sørensen R. Z., Christensen C. H., Nørskov J. K. (2008). Identification of Non-Precious Metal Alloy Catalysts for Selective Hydrogenation of Acetylene. Science.

[cit155] Studt F., Abild-Pedersen F., Bligaard T., Sørensen R. Z., Christensen C. H., Nørskov J. K. (2008). On the Role of Surface Modifications of Palladium Catalysts in the Selective Hydrogenation of Acetylene. Angew. Chem., Int. Ed..

[cit156] Feng Q., Zhao S., Wang Y., Dong J., Chen W., He D., Wang D., Yang J., Zhu Y., Zhu H., Gu L., Li Z., Liu Y., Yu R., Li J., Li Y. (2017). Isolated Single-Atom Pd Sites in Intermetallic Nanostructures: High Catalytic Selectivity for Semihydrogenation of Alkynes. J. Am. Chem. Soc..

[cit157] Spanjers C. S., Held J. T., Jones M. J., Stanley D. D., Sim R. S., Janik M. J., Rioux R. M. (2014). Zinc inclusion to heterogeneous nickel catalysts reduces oligomerization during the semi-hydrogenation of acetylene. J. Catal..

[cit158] Trimm D. L., Liu I. O. Y., Cant N. W. (2008). The oligomerization of acetylene in hydrogen over Ni/SiO2 catalysts: Product distribution and pathways. J. Mol. Catal. A: Chem..

[cit159] Ma J., Xing F., Nakaya Y., Shimizu K.-i., Furukawa S. (2022). Nickel-Based High-Entropy Intermetallic as a Highly Active and Selective Catalyst for Acetylene Semihydrogenation. Angew. Chem., Int. Ed..

[cit160] Nakaya Y., Hayashida E., Asakura H., Takakusagi S., Yasumura S., Shimizu K.-i., Furukawa S. (2022). High-Entropy Intermetallics Serve Ultrastable Single-Atom Pt for Propane Dehydrogenation. J. Am. Chem. Soc..

[cit161] Osswald J., Kovnir K., Armbrüster M., Giedigkeit R., Jentoft R. E., Wild U., Grin Y., Schlögl R. (2008). Palladium–gallium intermetallic compounds for the selective hydrogenation of acetylene: Part II: Surface characterization and catalytic performance. J. Catal..

[cit162] Prinz J., Pignedoli C. A., Stöckl Q. S., Armbrüster M., Brune H., Gröning O., Widmer R., Passerone D. (2014). Adsorption of Small Hydrocarbons on the Three-Fold PdGa Surfaces: The Road to Selective Hydrogenation. J. Am. Chem. Soc..

[cit163] Armbrüster M., Kovnir K., Friedrich M., Teschner D., Wowsnick G., Hahne M., Gille P., Szentmiklósi L., Feuerbacher M., Heggen M., Girgsdies F., Rosenthal D., Schlögl R., Grin Y. (2012). Al13Fe4 as a low-cost alternative for palladium in heterogeneous hydrogenation. Nat. Mater..

[cit164] Ledieu J., Gaudry É., Loli L. N. S., Villaseca S. A., de Weerd M. C., Hahne M., Gille P., Grin Y., Dubois J. M., Fournée V. (2013). Structural Investigation of the (010) Surface of the Al13Fe4 Catalyst. Phys. Rev. Lett..

[cit165] Niu Y., Huang X., Wang Y., Xu M., Chen J., Xu S., Willinger M.-G., Zhang W., Wei M., Zhang B. (2020). Manipulating interstitial carbon atoms in the nickel octahedral site for highly efficient hydrogenation of alkyne. Nat. Commun..

[cit166] Liu S., Li Y., Yu X., Han S., Zhou Y., Yang Y., Zhang H., Jiang Z., Zhu C., Li W.-X., Wöll C., Wang Y., Shen W. (2022). Tuning crystal-phase of bimetallic single-nanoparticle for catalytic hydrogenation. Nat. Commun..

[cit167] Zhou H., Yang X., Li L., Liu X., Huang Y., Pan X., Wang A., Li J., Zhang T. (2016). PdZn Intermetallic Nanostructure with Pd–Zn–Pd Ensembles for Highly Active and Chemoselective Semi-Hydrogenation of Acetylene. ACS Catal..

[cit168] Liu Y., Liu X., Feng Q., He D., Zhang L., Lian C., Shen R., Zhao G., Ji Y., Wang D., Zhou G., Li Y. (2016). Intermetallic NixMy (M = Ga and Sn) Nanocrystals: A Non-precious Metal Catalyst for Semi-Hydrogenation of Alkynes. Adv. Mater..

[cit169] Li D., Wang C., Tripkovic D., Sun S., Markovic N. M., Stamenkovic V. R. (2012). Surfactant Removal for Colloidal Nanoparticles from Solution Synthesis: The Effect on Catalytic Performance. ACS Catal..

[cit170] Ma J., Xing F., Shimizu K.-I., Furukawa S. (2024). Active site tuning based on pseudo-binary alloys for low-temperature acetylene semihydrogenation. Chem. Sci..

[cit171] Dasgupta A., Zimmerer E. K., Meyer R. J., Rioux R. M. (2019). Generalized approach for the synthesis of silica supported Pd-Zn, Cu-Zn and Ni-Zn gamma brass phase nanoparticles. Catal. Today.

[cit172] Armbrüster M., Wowsnick G., Friedrich M., Heggen M., Cardoso-Gil R. (2011). Synthesis and Catalytic Properties of Nanoparticulate Intermetallic Ga–Pd Compounds. J. Am. Chem. Soc..

[cit173] Cable R. E., Schaak R. E. (2007). Solution Synthesis of Nanocrystalline M−Zn (M = Pd, Au, Cu) Intermetallic Compounds via Chemical Conversion of Metal Nanoparticle Precursors. Chem. Mater..

[cit174] Onda A., Komatsu T., Yashima T. (2001). Preparation and Catalytic Properties of Single-Phase Ni–Sn Intermetallic Compound Particles by CVD of Sn(CH3)4 onto Ni/Silica. J. Catal..

[cit175] Kojima T., Nakaya Y., Ham H., Kameoka S., Furukawa S. (2021). Synthesis of Co2FeGe Heusler alloy nanoparticles and catalysis for selective hydrogenation of propyne. RSC Adv..

[cit176] Chen X., Kong C., Huang H., Chen D.-L., Wang F.-F., Zhang F., Zhu W. (2021). Mechanism of Selective Hydrogenation of 4-Nitrophenylacetylene Using Pt–Zn Intermetallic Nanoparticles: The Role of Hydrogen Coverage. J. Phys. Chem. C.

[cit177] Miyazaki M., Furukawa S., Komatsu T. (2017). Regio- and Chemoselective Hydrogenation of Dienes to Monoenes Governed by a Well-Structured Bimetallic Surface. J. Am. Chem. Soc..

[cit178] Sugiyama H., Miyazaki M., Sasase M., Kitano M., Hosono H. (2023). Room-Temperature CO2 Hydrogenation to Methanol over Air-Stable hcp-PdMo Intermetallic Catalyst. J. Am. Chem. Soc..

[cit179] Takayama T., Kariya R., Nakaya Y., Furukawa S., Yamazoe S., Komatsu T. (2021). Hydrosilylation of carbonyls over electron-enriched Ni sites of intermetallic compound Ni3Ga heterogeneous catalyst. Chem. Commun..

[cit180] Friedrich M., Teschner D., Knop-Gericke A., Armbrüster M. (2012). Influence of bulk composition of the intermetallic compound ZnPd on surface composition and methanol steam reforming properties. J. Catal..

